# PANX2 Suppresses Lung Adenocarcinoma Progression by Inducing Disulfidptosis and Enhancing Antitumor Immunity

**DOI:** 10.1002/advs.75662

**Published:** 2026-05-29

**Authors:** Yi Chen, Zhao‐Yu Liu, Gao‐Wen Qu, Run‐Hao Zeng, Wen‐Xia Yao, Yong‐Bing Zhou, Xue Liang, Yu‐Xiong Lai

**Affiliations:** ^1^ Key Laboratory of Biological Targeting Diagnosis Therapy and Rehabilitation of Guangdong Higher Education Institutes The Fifth Affiliated Hospital Guangzhou Medical University Guangzhou China; ^2^ Research Center of Cancer Diagnosis and Therapy Department of Oncology The First Affiliated Hospital of Jinan University Guangzhou China

**Keywords:** ATP signaling, cancer immunity, disulfidptosis, G6PD, lung adenocarcinoma, NRF2, PANX2

## Abstract

Lung adenocarcinoma (LUAD) remains a leading cause of cancer mortality with limited therapeutic options. Disulfidptosis, a novel cell death modality driven by disulfide stress, represents a promising target, yet its regulation in LUAD is poorly defined. Here, we identify Pannexin 2 (PANX2) as a tumor suppressor in LUAD. Clinically, PANX2 expression is downregulated during tumor progression, and low PANX2 levels correlate with poor prognosis. Functionally, PANX2 overexpression induces disulfidptosis in LUAD cells through two convergent axes: (i) activating NRF2 via Ca^2^^+^‐dependent stabilization and nuclear translocation to upregulate SLC7A11, increasing cystine uptake and NADPH consumption; (ii) suppressing G6PD to impair NADPH regeneration, compromising cystine clearance. This dual regulation synergistically depletes NADPH, causes cystine overload, and triggers disulfide crosslinking of cytoskeletal proteins, leading to actin cytoskeleton collapse. Moreover, PANX2 enhances extracellular ATP release, activating P2X7R signaling on immune cells to promote antitumor immune infiltration. In humanized mouse models, PANX2 overexpression suppresses tumor growth—effects reversed by NRF2 knockdown, G6PD overexpression, or P2X7R blockade. Collectively, our findings establish PANX2 as a tumor suppressor linking disulfidptosis to antitumor immunity, offering a dual‐target strategy for LUAD therapy.

## Graphical abstract

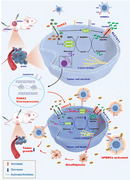

## 1 Introduction

Lung cancer remains the leading cause of cancer‐related mortality worldwide, with lung adenocarcinoma (LUAD) being the most prevalent histological subtype [1, 2]. Despite breakthroughs in targeted therapy and immunotherapy, intrinsic and acquired resistance often result in treatment failure [3, 4]. This highlights an urgent need to decipher novel molecular mechanisms underlying LUAD progression—particularly those involving metabolic reprogramming and dysregulated cell death pathways, which have emerged as actionable cancer vulnerabilities [5, 6].

Disulfidptosis, a recently identified form of programmed cell death, is distinct from apoptosis or ferroptosis and is characterized by the collapse of the actin cytoskeleton due to aberrant disulfide bond formation [7]. Cancer cells, driven by metabolic reprogramming and persistent oxidative stress, exhibit a strict dependency on cysteine to sustain glutathione (GSH)‐mediated antioxidant defense [8]. This dependency is met by two sequential processes: solute carrier family 7 member 11 (SLC7A11)‐mediated cystine import into the cell, followed by its NADPH‐dependent reduction to cysteine [9, 10]. Consequently, tumor cells typically display high cystine uptake and high cystine reduction rates. However, this very NADPH‐dependent metabolic pattern renders them vulnerable to disulfidptosis under stress conditions. When NADPH is depleted—triggered by glucose starvation or oxidative stress—the clearance of excess disulfides (primarily cystine) is impaired in SLC7A11‐high cells, leading to pathological disulfide crosslinking of actin cytoskeletal proteins (disulfide stress) [11, 12]. This disrupts cytoskeletal architecture, manifesting as filamentous actin (F‐actin) contraction and membrane‐cytoskeleton dissociation, and ultimately causes cell death. Clinical analyses have revealed that disulfidptosis levels are lower in various cancers, including LUAD, and that higher intratumoral disulfidptosis correlates with a favorable prognosis and enhanced antitumor immune infiltration [13, 14, 15, 16]. Despite its emerging significance, the upstream regulators of disulfidptosis in LUAD—particularly the interplay between metabolic enzymes and membrane channels—remain poorly characterized.

The Pannexin family comprises three structurally similar transmembrane channel proteins—Pannexin 1 (PANX1), 2 (PANX2), and 3 (PANX3)—that form oligomeric pores in the cell membrane, mediating the transport of molecules such as ATP and Ca^2^^+^ ions to facilitate intracellular and intercellular communication [17, 18, 19]. Studies have shown that PANX1 regulates apoptosis and pyroptosis, with specific modulators like carbenoxolone and probenecid demonstrating therapeutic potential in diseases and tumors [20]. Genetic or pharmacological inhibition of PANX1 alleviates inflammation, suppresses immune responses, and improves pathological phenotypes in inflammatory disease models [21], highlighting the Pannexin family's dual role in cell death regulation and immune modulation. In contrast, PANX2's functions and mechanisms remain poorly understood, with existing studies focusing on its glioma‐suppressive role while its function in lung cancer remains largely unexplored [22, 23]. Notably, PANX2's channel function enables it to regulate Ca^2^^+^ flux and ATP release—two critical modulators of redox metabolism that dictate cancer cell susceptibility to stress‐induced death, while extracellular ATP (eATP) simultaneously shapes immune cell function [24, 25, 26]. This eATP serves as a key danger‐associated molecular pattern (DAMP) that activates immune cells primarily through P2 receptors (P2R), particularly P2X7R, a major ATP sensor on immune cells that promotes inflammatory and cytotoxic responses [27, 28].

Given these links, in this study, we aimed to investigate the clinical significance, biological function, and underlying mechanisms of PANX2 in LUAD, focusing on its potential involvement in cell death regulation and tumor immunology.

## 2 Results

### 2.1 PANX2 Expression Decreases With LUAD Progression and Correlates With Poor Prognosis

**FIGURE 1:**
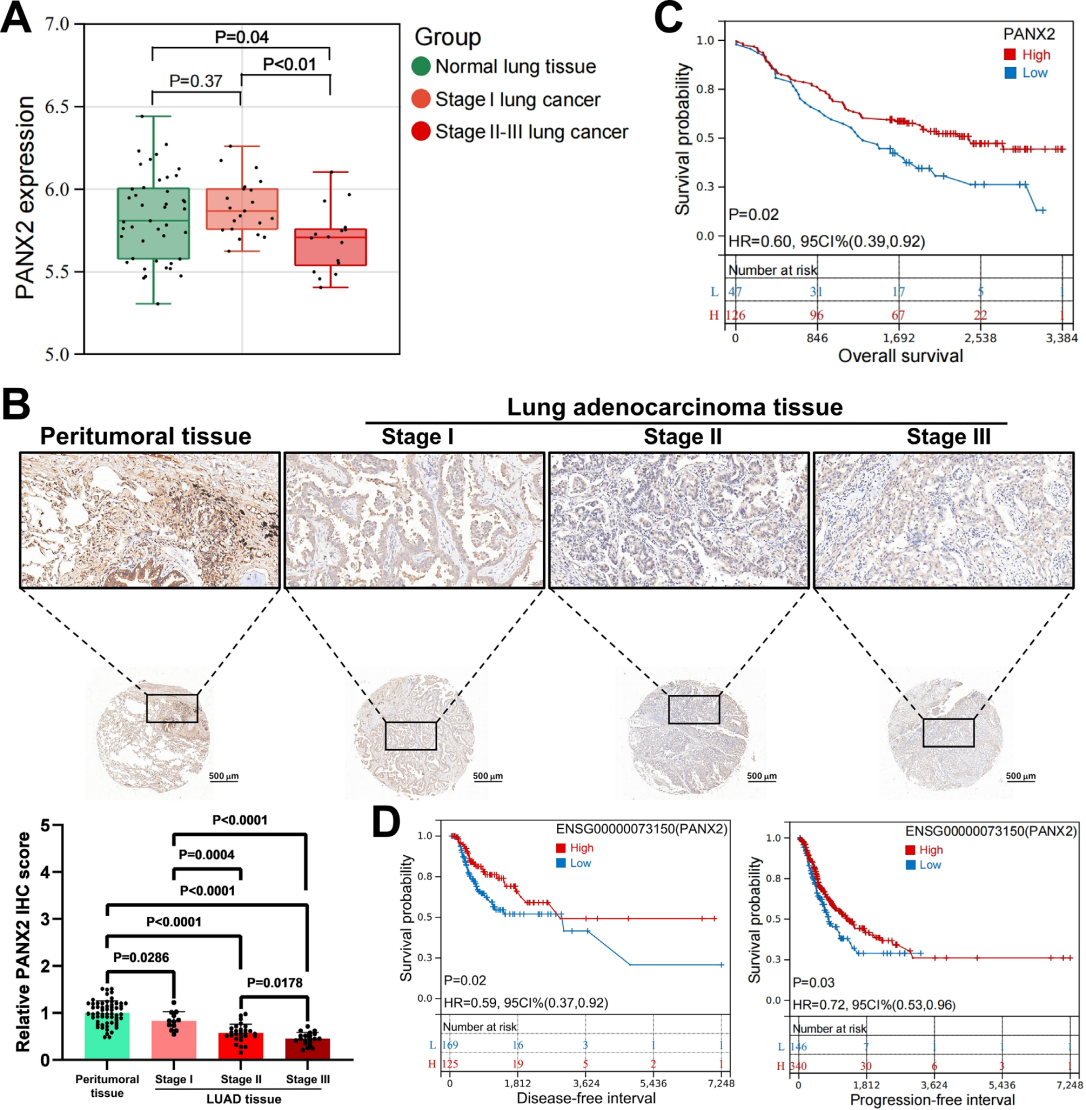
PANX2 expression decreases with LUAD progression and correlates with poor prognosis. (A) GSE40275 dataset analysis of PANX2 expression across clinical stages. (B) IHC quantification of PANX2 protein in TMA by clinical stage. (C) OS analysis by high/low‐PANX2 expression in LUAD (HPA). (D) DFI and PFI analyses by high/low‐PANX2 expression in LUAD (TCGA). (Data are presented as means ± SD).

To evaluate the clinical significance of PANX2 in LUAD, we first performed an exploratory analysis of transcriptome data from the Gene Expression Omnibus (GEO) database (GSE40275), comprising 43 normal lung tissues and 37 primary lung cancer tissues (excluding 4 metastatic cases). PANX2 expression was significantly downregulated in Stage II‐III (intermediate and locally advanced stages) tumors compared with Stage I (early‐stage) tumors (*p* < 0.05) (Figure 1A). To validate this at the protein level, we performed immunohistochemistry (IHC) on a LUAD‐specific tissue microarray (TMA) containing 60 paired tumor and adjacent normal tissues. Quantitative analysis confirmed that the PANX2 protein expression intensity in LUAD tissues was significantly lower than that in matched adjacent normal tissues, and its expression exhibited a stepwise decrease with advancing clinical stages (Stage I, *n* = 12; Stage II, *n* = 28; Stage III, *n* = 20; *p* < 0.05) (Figure 1B).

Survival analysis further underscored the prognostic value of PANX2. Integration of data from Human Protein Atlas (HPA, *n* = 173) and The Cancer Genome Atlas (TCGA, *n* = 490) databases revealed that low PANX2 expression was significantly associated with poorer overall survival (OS) (HR > 1, log‐rank *p* < 0.05) (Figure 1C). Additionally, patients with high PANX2 expression had remarkably prolonged disease‐free interval (DFI) and progression‐free interval (PFI) (HR < 1, log‐rank *p* < 0.05) (Figure 1D). Collectively, these data suggest that PANX2 may function as a tumor suppressor in LUAD.

### 2.2 PANX2 Overexpression Suppresses LUAD Malignancy via Disulfide Bond Stress

**FIGURE 2:**
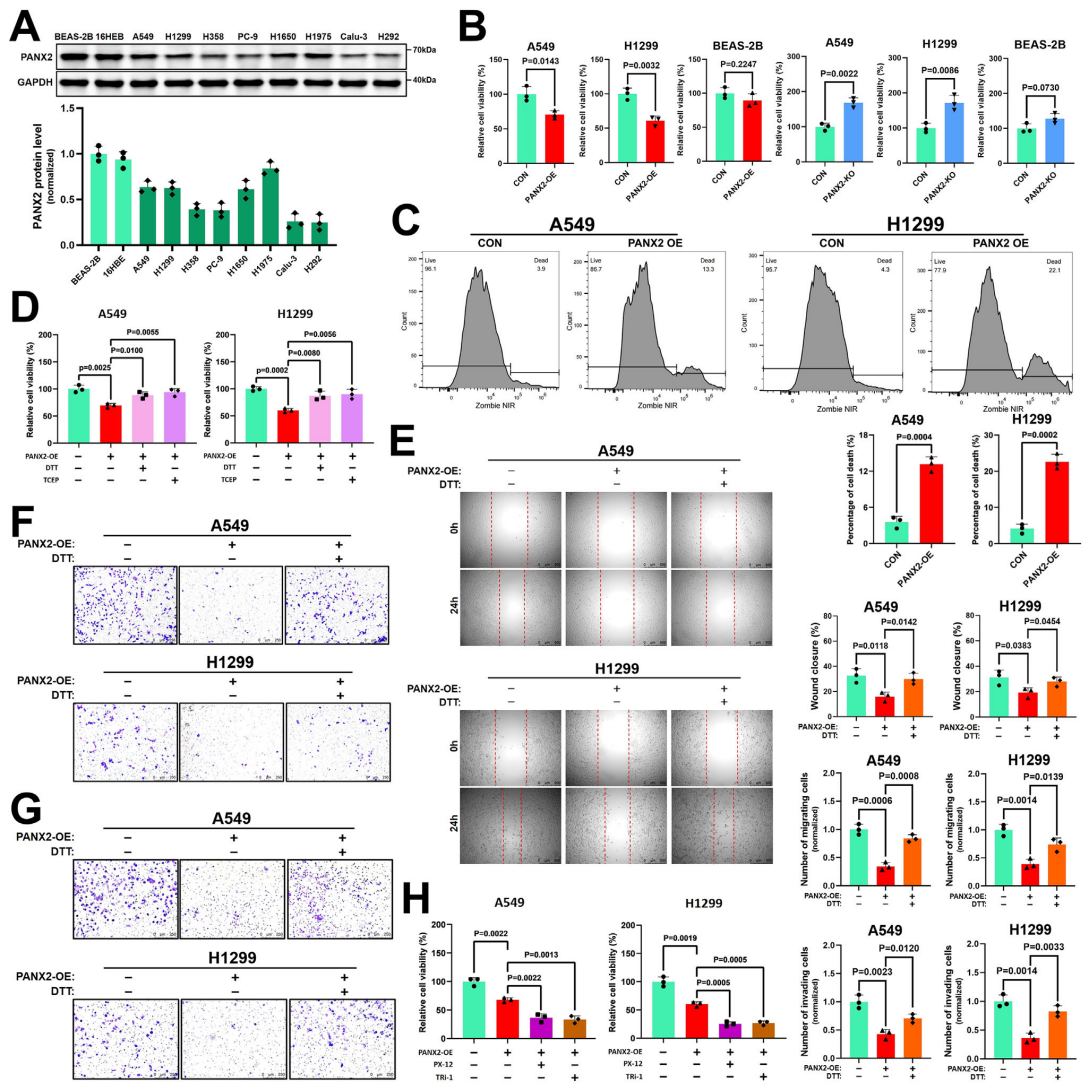
PANX2 overexpression suppresses LUAD malignancy via disulfide bond stress. (A) WB analysis of PANX2 protein levels in diverse cell lines. (B) CCK‐8 assay of cell viability in PANX2‐OE/KO cells vs. respective controls. (C) Flow cytometry analysis of PANX2‐OE‐induced cell death in A549/H1299 cells. (D) CCK‐8 assay: DTT (2 mM)/TCEP (1 mM) rescued PANX2‐OE‐induced viability loss in LUAD cells, 24 h. (E) Scratch assay: DTT (2 mM, 24 h) rescued PANX2‐OE‐impaired cell migration in LUAD cells. (F) Transwell migration assay: DTT (2 mM, 24 h) rescued PANX2‐OE‐impaired cell migration in LUAD cells. (G) Transwell invasion assay: DTT (2 mM, 24 h) rescued PANX2‐OE‐impaired cell invasion in LUAD cells. (H) CCK‐8 assay: PX12/TRi‐1 (10 µM) exacerbated PANX2‐OE‐induced viability loss in LUAD cells, 24 h. (Data are presented as means ± SD; *n* ≥ 3 independent experiments; “OE,” “KO,” and “CON” denote overexpression, knockout, and control, respectively).

Consistent with the clinical findings, PANX2 expression was generally lower at both transcriptional and protein levels in a panel of 8 LUAD cell lines (A549, H1299, H358, PC‐9, H1650, H1975, Calu‐3, and H292) than in normal lung epithelial cell lines (BEAS‐2B and 16HBE) (Figure 2A and Figure S1A). To investigate its biological function, we established stable PANX2 overexpression (PANX2‐OE) and CRISPR/Cas9‐mediated knockout (PANX2‐KO) models in A549, H1299, and BEAS‐2B cells, with efficiency verified by Western blot (WB) and qRT‐PCR (Figure S1B,C).

Functional analyses revealed that PANX2‐OE significantly inhibited the proliferation, colony formation, migration, and invasion of LUAD cells, whereas PANX2‐KO promoted these malignant phenotypes (Figure S1D–H). Microscopic observation showed that PANX2‐OE cells exhibited morphological alterations, including rounding and poor adhesion, while PANX2‐KO cells appeared more spread (Figure S2A). Accordingly, PANX2‐OE reduced cell viability and increased cell death, as assessed by CCK‐8 assay and flow cytometry, with opposing effects observed upon PANX2‐KO (Figure 2B,C). No such effects were observed in BEAS‐2B cells.

Gene set enrichment analysis (GSEA) on the exploratory GSE40275 dataset indicated that samples with high PANX2 expression were enriched in pathways related to actin cytoskeleton polymerization, immune response, and redox reactions (Figure S2B–E). These trends were consistently validated in subsequent LUAD cell models and functional experiments, guiding our investigations into disulfide bond stress and immune modulation

To identify the death modality, pharmacological rescue experiments showed that inhibitors of apoptosis (Z‐VAD‐FMK), necroptosis (necrostatin‐1, Nec‐1), autophagy (chloroquine, CQ), pyroptosis (AC‐YVAD‐CMK), or ferroptosis (ferrostatin‐1, Fer‐1)  all failed to rescue the viability loss induced by PANX2‐OE (Figure S2F) [29]. The efficacy of each inhibitor was confirmed via positive control assays (Figure S2G), and PANX2‐OE did not alter the levels of key executioner proteins for these cell death types (Figure S2H). We also ruled out energy depletion and oxidative stress as primary causes, as intracellular ATP levels were maintained and the ROS scavenger NAC failed to rescue cell death despite increased ROS (Figure S2I–K) [30, 31].

Conversely, the disulfide bond reducer dithiothreitol (DTT) or tris(2‐chloroethyl) phosphate (TCEP) not only rescued the cell viability loss (Figure 2D) but also partially reversed the suppression of migration and invasion (Figure 2E–G) [32]. In contrast, thioredoxin system inhibitors (PX‐12 or TRi‐1) exacerbated cell death (Figure 2H) [33, 34]. These data strongly suggest that PANX2 regulates LUAD malignancy by inducing disulfide bond stress, pointing to disulfidptosis.

### 2.3 PANX2 Triggers Disulfidptosis via SLC7A11‐Mediated Cystine Overload and Actin Crosslinking

**FIGURE 3:**
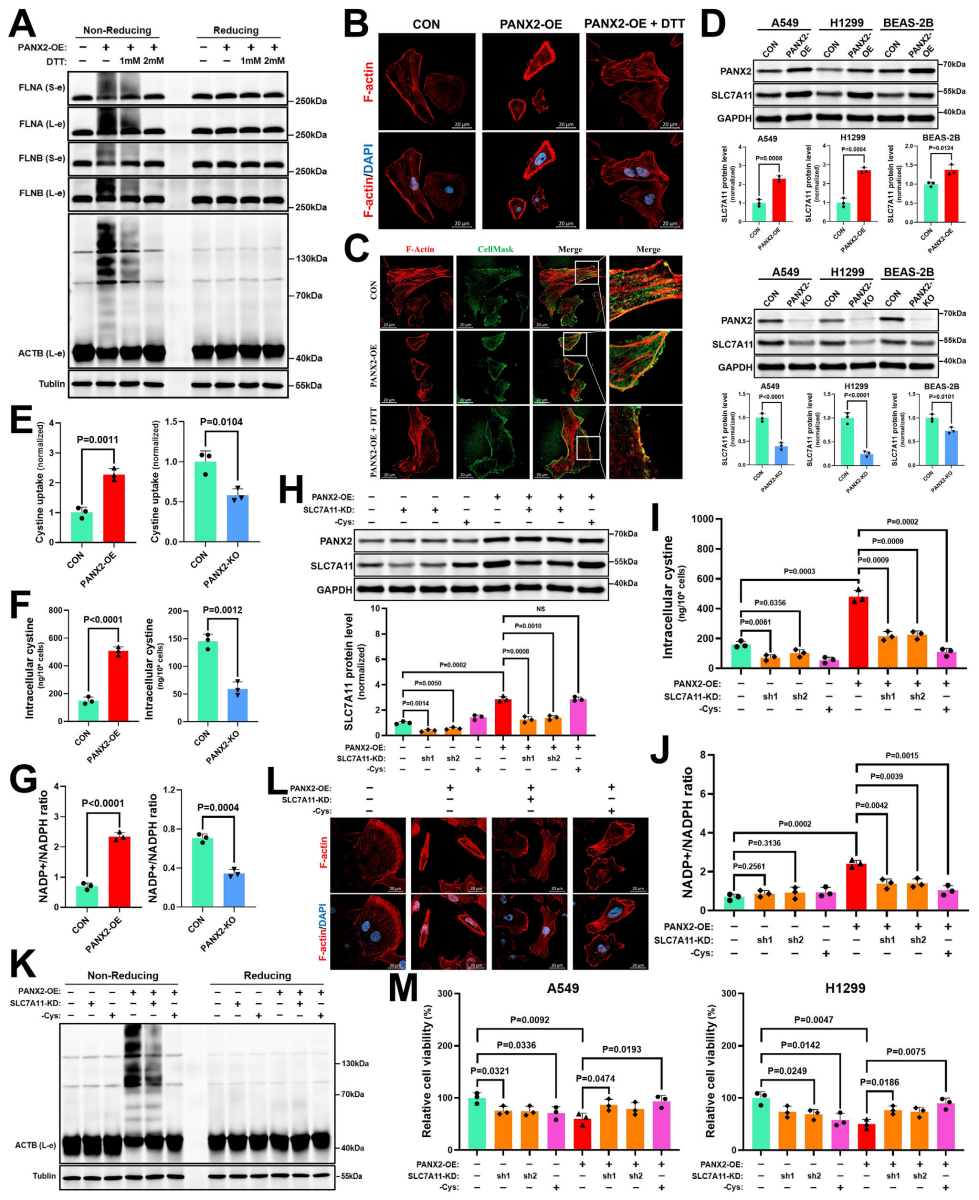
PANX2 triggers disulfidptosis via SLC7A11‐mediated cystine overload and actin crosslinking. (A) Nonreducing SDS‐PAGE: PANX2‐OE induces disulfide crosslinking of cytoskeletal proteins in A549 cells, and DTT (2 mM, 24 h) reverses this effect. Short exposure (S‐e) shows monomeric bands; long exposure (L‐e) reveals crosslinked aggregates. (B) Phalloidin staining: PANX2‐OE induces F‐actin contraction in A549 cells and DTT (2 mM, 24 h) reverses this effect. (C) Phalloidin and DiO co‐staining: PANX2‐OE induces disruption of cytoskeleton‐membrane interactions in A549 cells and DTT (2 mM, 24 h) reverses this effect. (D) WB to evaluate SLC7A11 protein levels in PANX2‐OE/KO cells vs. respective controls. (E) Cystine uptake assay in PANX2‐OE/KO A549 cells vs. controls. (F) Cystine level detection in PANX2‐OE/KO A549 cells vs. controls. (G) NADP^+^/NADPH ratio analysis of NADPH consumption in PANX2‐OE/KO A549 cells vs. controls. (H) WB to assess the effect of SLC7A11‐KD or cystine starvation on PANX2‐OE‐upregulated SLC7A11 protein levels in A549 cells. (I) Cystine level detection: SLC7A11‐KD or cystine starvation (‐Cys) reverses PANX2‐OE‐upregulated cystine levels in A549 cells. (J) NADP+/NADPH ratio analysis: SLC7A11‐KD or ‐Cys reverses PANX2‐OE‐upregulated NADPH consumption in A549 cells. (K) Nonreducing SDS‐PAGE: SLC7A11‐KD or ‐Cys reversed PANX2‐OE‐induced disulfide crosslinking of cytoskeletal proteins in A549 cells (Long exposure). (L) Phalloidin staining: SLC7A11‐KD or ‐Cys reversed PANX2‐OE‐induced F‐actin contraction in A549 cells. (M) CCK‐8 assay: SLC7A11‐KD or ‐Cys rescued PANX2‐OE‐induced viability loss in LUAD cells. (Data are presented as means ± SD; *n* ≥ 3 independent experiments; “OE,” “KO,” “KD,” and “CON” denote overexpression, knockout, knockdown, and control, respectively).

To define the cell death modality induced by PANX2, we focused on the hallmark feature of disulfidptosis: pathological disulfide crosslinking of cytoskeletal proteins. Non‐reducing SDS‐PAGE analysis (with N‐ethylmaleimide to preserve disulfide bonds) revealed extensive high‐molecular‐weight smearing of key cytoskeletal components—the actin‐crosslinking proteins filamin A/B (FLNA/B) and the actin monomer β‐actin (ACTB)—in PANX2‐OE LUAD cells [11, 12], indicative of intermolecular disulfide bond formation (Figure 3A and Figure S3A). This abnormal crosslinking was completely reversed by the reducing agent DTT. Consistently, phalloidin staining showed that PANX2‐OE LUAD cells, but not PANX2‐OE BEAS‐2B cells, exhibited severe F‐actin contraction and actin‐membrane detachment (Figure 3B,C and Figure S3B,C). Crucially, DTT treatment reversed the protein aggregation, restored F‐actin architecture, and rescued cell viability (Figure 2D), directly linking disulfide stress to cell death and confirming PANX2 induces disulfidptosis in LUAD cells.

We next investigated the upstream trigger of this disulfide overload. Since disulfidptosis is initiated by cystine accumulation coupled with NADPH depletion, we examined the cystine transporter SLC7A11. Consistent with a positive correlation observed in lung cancer transcriptomes (Figure S3D). PANX2‐OE in LUAD cells upregulated SLC7A11 at both transcriptional and protein levels (Figure 3D and Figure S3E), leading to enhanced cystine uptake and elevated intracellular cystine concentration (Figure 3E,F and Figure S3F,G). The increased cystine flux imposes a reductive burden, consuming NADPH for its conversion to cysteine. Accordingly, PANX2‐OE cells exhibited a marked elevation in the NADP^+^/NADPH ratio (Figure 3G and Figure S3H), indicating severe NADPH depletion and impaired cellular reductive capacity. Notably, these PANX2‐induced alterations were minimal in BEAS‐2B cells, underscoring the pathway's specificity in LUAD.

To validate the necessity of SLC7A11‐mediated cystine overload in this pathway, we performed loss‐of‐function rescue experiments. Stable knockdown (KD) of SLC7A11 (shSLC7A11) in PANX2‐OE cells reversed the increases in cystine level and NADPH depletion, dissolved the cytoskeletal protein aggregates, and restored F‐actin architecture (Figure 3H–M and Figure S3I–N). Notably, in contrast to its inhibitory effect on the viability of control cells, SLC7A11‐KD rescued viability in PANX2‐OE cells, highlighting the specific dependency of PANX2‐induced death on cystine flux [11, 12]. Furthermore, cystine starvation recapitulated this comprehensive rescue, confirming cystine overload as a prerequisite for PANX2‐induced disulfidptosis (Figure 3H–M and Figure S3I–N).

### 2.4 PANX2 Activates NRF2 to Drive SLC7A11 Expression and Disulfidptosis

**FIGURE 4:**
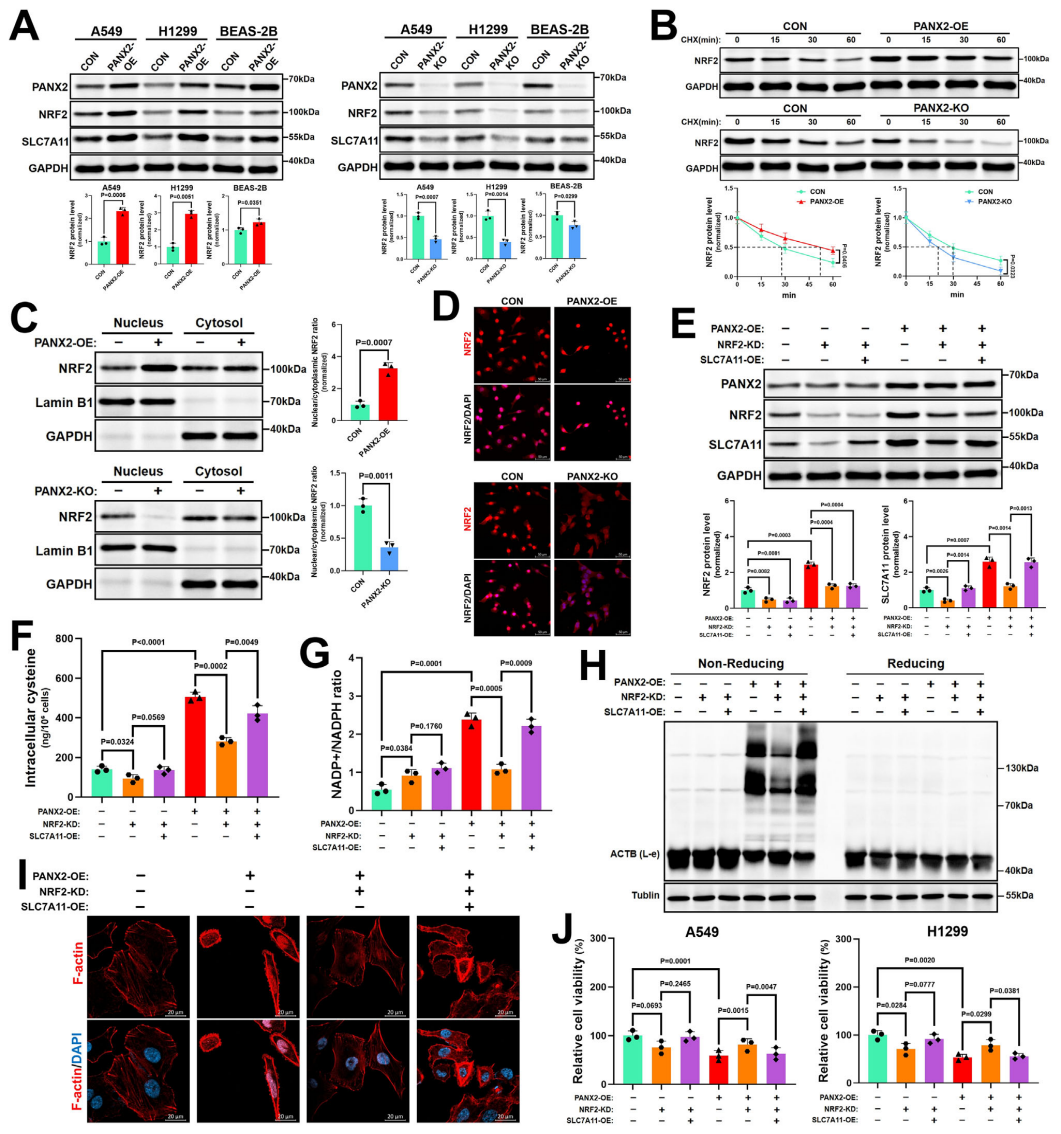
PANX2 activates NRF2 to drive SLC7A11 expression and disulfidptosis. (A) WB to evaluate NRF2 protein levels in PANX2‐OE/KO cells vs. controls. (B) WB time‐course analysis of NRF2 protein stability upon PANX2‐OE/KO in CHX‐treated A549 cells (40 µg/ml). (C) Nuclear‐cytoplasmic fractionation WB to assess NRF2 protein levels in the nucleus and cytoplasm upon PANX2‐OE/KO in A549 cells. (D) Immunofluorescence staining to assess NRF2 nuclear translocation upon PANX2‐OE/KO in A549 cells. (E) WB to assess the effect of NRF2‐KD and subsequent SLC7A11 re‐expression on PANX2‐OE‐upregulated NRF2 and SLC7A11 protein levels in A549 cells. (F) Cystine level detection to assess the effect of NRF2‐KD and subsequent SLC7A11 re‐expression on PANX2‐OE‐upregulated cystine level in A549 cells. (G) NADP+/NADPH ratio analysis to assess the effect of NRF2‐KD and subsequent SLC7A11 re‐expression on PANX2‐OE‐upregulated NADPH consumption in A549 cells. (H) Nonreducing SDS‐PAGE assay to assess the effect of NRF2‐KD and subsequent SLC7A11 re‐expression on PANX2‐OE‐induced disulfide crosslinking of cytoskeletal proteins in A549 cells (Long exposure). (I) Phalloidin staining assay to assess the effect of NRF2‐KD and subsequent SLC7A11 re‐expression on PANX2‐OE‐induced F‐actin contraction in A549 cells. (J) CCK‐8 assay to assess the effect of NRF2‐KD and subsequent SLC7A11 re‐expression on PANX2‐OE‐induced viability loss in LUAD cells. (Data are presented as means ± SD; *n* ≥ 3 independent experiments; “OE,” “KO,” “KD,” and “CON” denote overexpression, knockout, knockdown, and control, respectively.

We next sought to determine how PANX2 upregulates SLC7A11. Prompted by a correlative link between PANX2 and nuclear factor erythroid 2–related factor 2 (NRF2, NFE2L2)—a key transcription factor for SLC7A11—in lung cancer transcriptomes (Figure S4A) [35], PANX2‐OE increased, while PANX2‐KO decreased, NRF2 protein levels in LUAD cells, with minimal effect in BEAS‐2B cells (Figure 4A). Notably, PANX2‐OE did not alter NRF2 mRNA abundance, suggesting post‐transcriptional regulation (Figure S4B).

To delineate the mechanism, we assessed NRF2 protein stability and subcellular localization. Cycloheximide chase assay showed that PANX2‐OE markedly prolonged NRF2 protein half‐life, whereas PANX2‐KO shortened it (Figure 4B and Figure S4C). Furthermore, subcellular fractionation and immunofluorescence confirmed enhanced NRF2 nuclear accumulation in PANX2‐OE cells (Figure 4C,D). PANX2‐OE also elevated the mRNA levels of canonical NRF2 transcriptional targets NQO1 and HMOX1 (Figure S4D), further corroborating that PANX2 activates the NRF2 pathway. Intriguingly, PANX2‐OE increased intracellular Ca^2+^ flux, consistent with its channel function (Figure S4E). This supports a Ca^2^^+^‐dependent mechanism for PANX2‐mediated NRF2 activation, as BAPTA‐AM‐mediated Ca^2^^+^ chelation reversed the PANX2‐OE‐induced upregulation of NRF2 (Figure S4F) [36, 37, 38, 39].

To establish the functional necessity of NRF2, we knocked it down in PANX2‐OE cells. This reversed the upregulation of SLC7A11, intracellular cystine levels, NADPH depletion, cytoskeletal disulfide crosslinking, F‐actin contraction, and cell death (Figure 4E–J and Figure S4G–L). Crucially, re‐expressing SLC7A11 in the NRF2‐KD background restored cystine overload and disulfidptosis phenotypes (Figure 4E–J and Figure S4G–L), confirming NRF2 acts primarily through SLC7A11. Together, these results define a PANX2/NRF2/SLC7A11/cystine pathway that drives disulfidptosis in LUAD.

### 2.5 PANX2 Suppresses G6PD to Exacerbate NADPH Depletion and Synergistically Promote Disulfidptosis

Since cellular reductive capacity, determined by NADPH levels, critically influences susceptibility to disulfide stress, we investigated whether PANX2 regulates NADPH generation independent of cystine uptake. NADPH is primarily generated from glucose, which is taken up by glucose transporters, particularly GLUT1 (SLC2A1) and GLUT3 (SLC2A3), and subsequently metabolized through the pentose phosphate pathway (PPP) [40]. Bioinformatic and experimental analyses first excluded glucose uptake as a target, showing no correlation between PANX2 and glucose transporter expression and no effect of PANX2‐OE on glucose uptake capacity in LUAD cells (Figure S5A–E).

**FIGURE 5:**
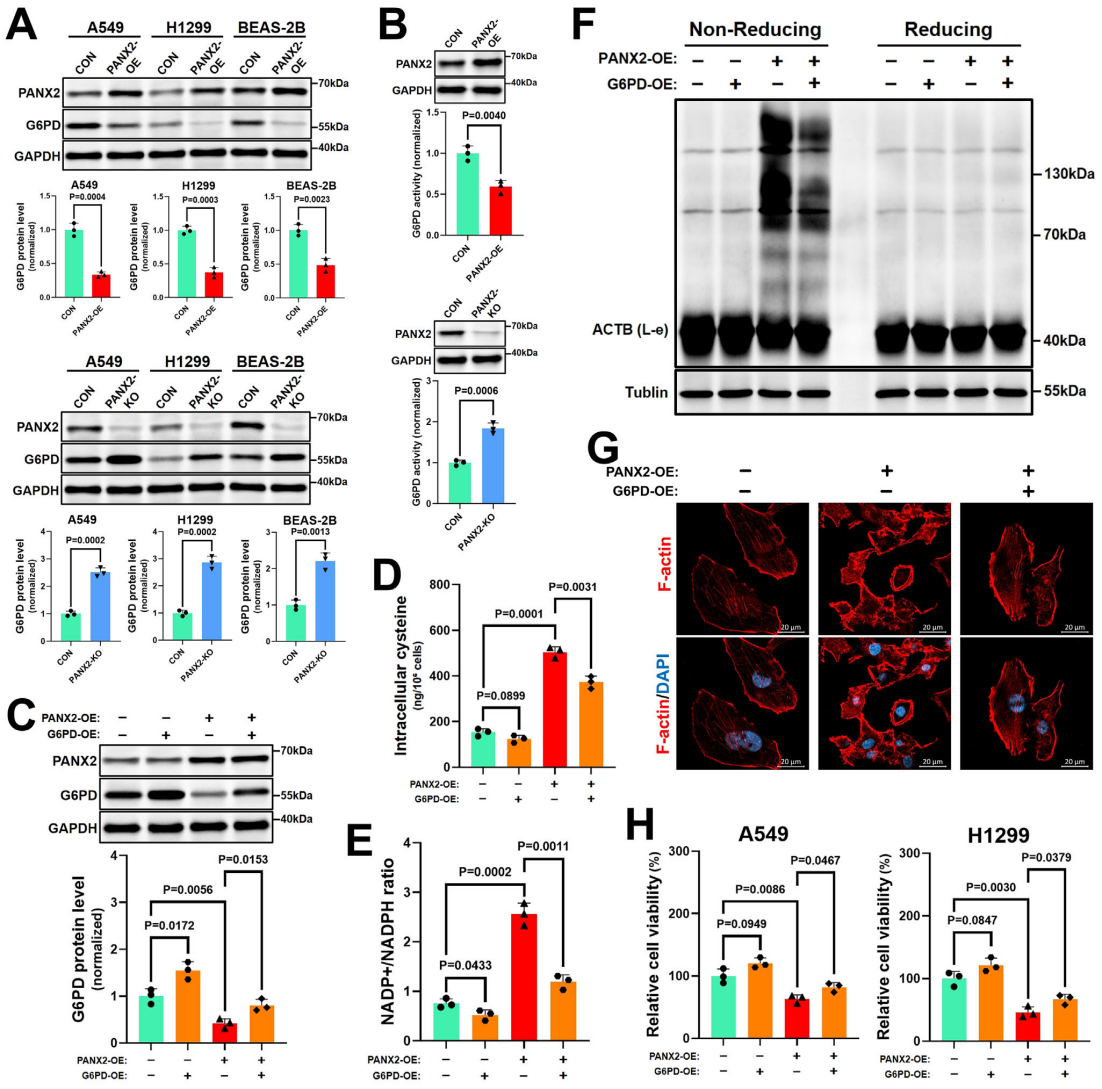
PANX2 suppresses G6PD to exacerbate NADPH depletion and synergistically promote disulfidptosis. (A) WB to evaluate G6PD protein levels in PANX2‐OE/KO cells vs. controls. (B) G6PD activity assay in PANX2‐OE/KO A549 cells vs. respective controls. (C) WB to assess the effect of G6PD‐OE on PANX2‐OE‐downregulated G6PD protein levels in A549 cells. (D) Cystine level detection: G6PD‐OE reverses PANX2‐OE‐upregulated cystine levels in A549 cells. (E) NADP+/NADPH ratio analysis: G6PD‐OE reverses PANX2‐OE‐upregulated NADPH consumption in A549 cells. (F) Nonreducing SDS‐PAGE: G6PD‐OE reversed PANX2‐OE‐induced disulfide crosslinking of cytoskeletal proteins in A549 cells (Long exposure). (G) Phalloidin staining: G6PD‐OE reversed PANX2‐OE‐induced F‐actin contraction in A549 cells. (H) CCK‐8 assay: G6PD‐OE rescued PANX2‐OE‐induced viability loss in LUAD cells. (Data are presented as means ± SD; *n* ≥ 3 independent experiments; “OE,” “KO,” and “CON” denote overexpression, knockout, and control, respectively).

We therefore focused on the PPP. A significant negative correlation was observed between PANX2 and the expression of glucose‐6‐phosphate dehydrogenase (G6PD), the PPP rate‐limiting enzyme (Figure S5A) [41]. Functionally, PANX2‐OE downregulated G6PD mRNA and protein levels and suppressed its enzymatic activity, while PANX2‐KO had the opposite effects (Figure 5A,B and Figure S5F,G). This suppression led to impaired NADPH regeneration (elevated NADP^+^/NADPH ratio) and a consequent exacerbation of intracellular cystine accumulation (Figure 3F,G and Figure S3G,H), resulting in more severe cytoskeletal disulfide crosslinking and F‐actin network collapse in LUAD cells (Figure 3A,B and Figure S3A,B). Conversely, PANX2‐KO cells maintained higher G6PD activity and NADPH reserves, which conferred resistance to disulfide stress.

The necessity of G6PD inhibition was confirmed by rescue experiments. G6PD‐OE in PANX2‐OE cells restored NADPH levels, reduced cystine accumulation, reversed cytoskeletal damage, and partially rescued cell viability (Figure 5C–H and Figure S5H–M). Notably, PANX2‐OE‐mediated suppression of G6PD persisted upon NRF2‐KD, indicating regulation independent of the NRF2‐SLC7A11 pathway (Figure S5N). These results define a PANX2/G6PD/NADPH/cystine pathway that compromises cystine clearance.

To assess its synergy with the PANX2/NRF2/SLC7A11/cystine axis, we performed combinatorial interventions. Co‐treatment with Sulfasalazine (SAS, an SLC7A11 inhibitor) or ML385 (an NRF2 inhibitor) and AG1 (a G6PD activator) reversed PANX2‐OE‐induced cell death more effectively than any single agent (Figure S5O) [42, 43, 44]. Conversely, concomitant NRF2‐OE and G6PD‐KD in control cells mimicked the cell death induced by PANX2‐OE (Figure S5P). Collectively, PANX2 drives disulfidptosis through two convergent metabolic stresses: upregulated cystine import and impaired NADPH‐dependent cystine reduction.

### 2.6 PANX2 Overexpression in LUAD Cells Activates Antitumor Immunity via eATP‐P2X7R Signaling

Bioinformatic analysis of lung cancer transcriptomes suggested an immunomodulatory role for PANX2, as single‐sample gene set enrichment analysis (ssGSEA) revealed that its high expression correlated with increased infiltration of multiple antitumor immune cells—including CD8^+^ T lymphocytes (T cells), natural killer (NK) cells, dendritic (DC) cells, B lymphocytes (B cells), and macrophages (Figure S6A). To validate these predictions in a LUAD context, we established a direct co‐culture model of A549 cells (PANX2‐OE, PANX2‐KO, or controls) with human peripheral blood mononuclear cells (hPBMCs) (Figure S6B) [45, 46, 47]. Microscopic observation showed enhanced hPBMC aggregation around PANX2‐OE A549 cells, which was reduced in the PANX2‐KO group (Figure S6C).

**FIGURE 6:**
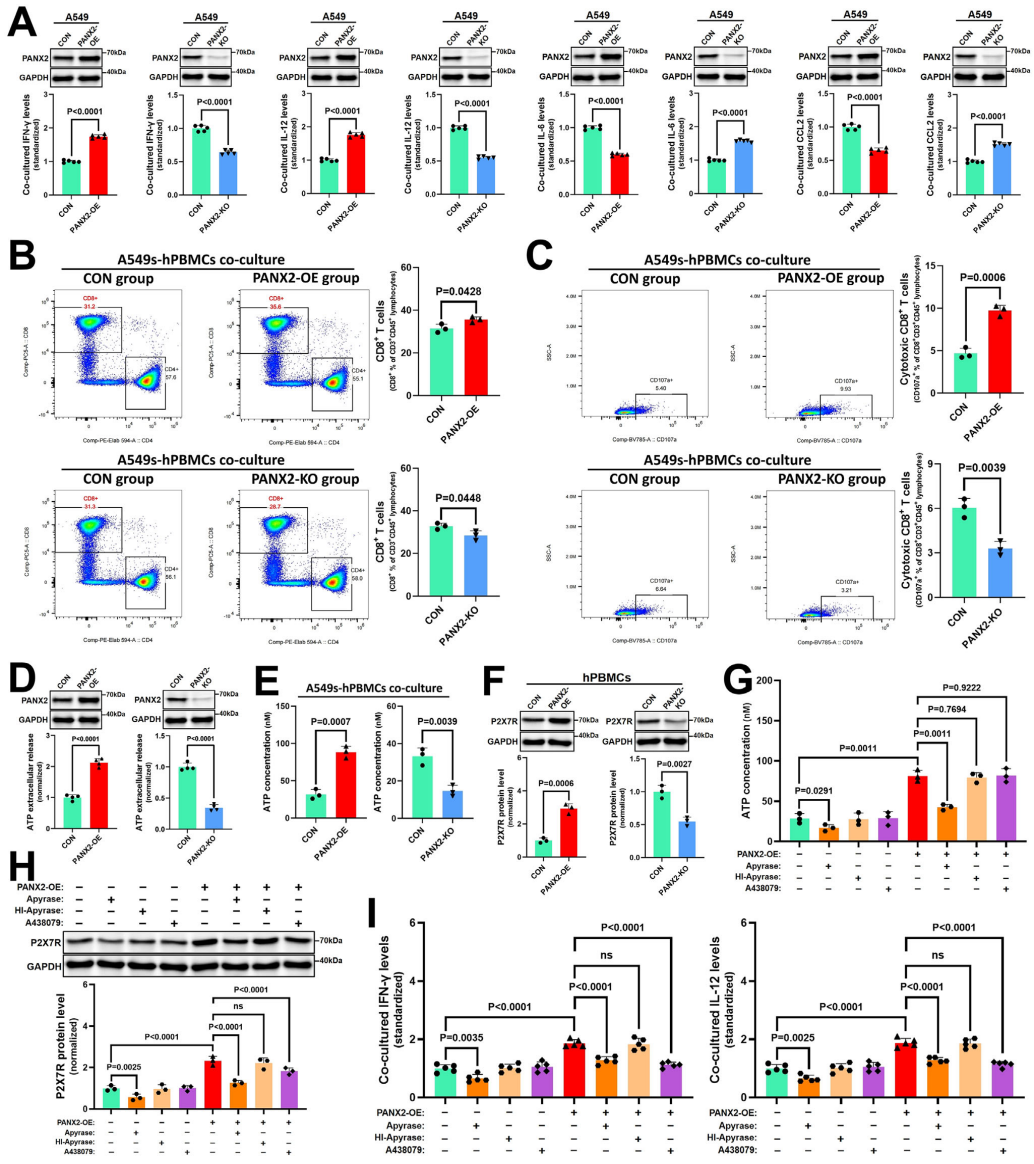
PANX2 overexpression in LUAD cells activates antitumor immunity via eATP‐P2X7R signaling. (A) ELISA of cytokines in co‐culture supernatants from PANX2‐OE/KO co‐cultures vs. respective controls. (B) Flow cytometry immunophenotyping: percentage of CD8^+^ T cells in total T cells from PANX2‐OE/KO co‐cultures vs. controls. (C) Flow cytometry: percentage of CD107a^+^ cytotoxic subset in CD8^+^ T cells from PANX2‐OE/KO co‐cultures vs. controls. (D) Chemiluminescence assay of extracellular ATP release in PANX2‐OE/KO A549 cells vs. controls. (E) Chemiluminescence assay of ATP concentration modulation by PANX2‐OE/KO in A549 cells within the co‐culture microenvironment vs. controls. (F) WB to assess P2X7R protein levels in hPBMCs co‐cultured with PANX2‐OE/KO A549 cells vs. controls. (G) ATP assay to evaluate the reversal effect of Apyrase (10 U/mL) or A438079 (10 µM) on PANX2‐OE‐increased ATP levels in co‐cultures after 72 h (heat‐inactivated Apyrase as control). (H) WB: Apyrase (10 U/mL) or A438079 (10 µM) reverses PANX2‐OE‐increased P2X7R protein levels in hPBMCs after 72 h co‐culture (heat‐inactivated (HI)‐Apyrase as control). (I) ELISA: Apyrase (10 U/mL) or A438079 (10 µM) reverses PANX2‐OE‐increased cytokine secretion in co‐cultures (HI‐Apyrase as control). (Data are presented as means ± SD; *n* ≥ 3 independent experiments; “OE,” “KO,” and “CON” denote overexpression, knockout, and control, respectively).

Enzyme‐linked immunosorbent assay (ELISA) of co‐culture supernatants demonstrated that PANX2‐OE significantly enhanced the secretion of key immunostimulatory cytokines (e.g., IFN‐γ, IL‐12), while suppressing immunosuppressive factors (e.g., IL‐6) (Figure 6A and Figure S6D) [48, 49, 50, 51, 52, 53, 54]. Flow cytometry immunophenotyping further confirmed that PANX2‐OE increased the proportion of CD8^+^ T cells (CD8^+^/CD3^+^CD45^+^ lymphocytes) and their cytotoxic subpopulation (CD107a^+^/CD8^+^CD3^+^CD45^+^ lymphocytes) (Figure 6B,C) [55, 56], elevated the percentage of total NK cells (CD56^+^/CD3^−^CD45^+^ lymphocytes) and promoted their polarization toward the highly cytotoxic CD56dimCD16^+^ subpopulation (CD56dimCD16^+^/CD56briCD16^−^) [57, 58], whereas PANX2‐KO exerted the opposite effects (Figure S6E,F). Additionally, it increased the proportions of DC cells (CD11c^+^/CD45^+^ monocytes), B cells (CD19^+^/CD3^−^CD45^+^ lymphocytes), and M1 macrophages (CD86^+^CD206^−^/CD14^+^CD45^+^ monocytes), inducing M1 macrophage polarization (CD86^+^CD206^−^/CD206^+^CD86^−^ of CD14^+^ monocytes)—effects that were consistently reduced in the PANX2‐KO group (Figure S6G–I) [59, 60, 61]. Together, these findings indicate that PANX2‐OE in LUAD cells promotes antitumor immune cell activation, proliferation, and recruitment, suggesting potential to reshape the tumor immune microenvironment and elicit enhanced antitumor immunity.

To delineate the mechanism underlying PANX2‐mediated immune activation, we focused on its ATP‐permeable channel function. We hypothesized that PANX2‐OE boosts eATP release via increased membrane channel permeability and disulfidptosis‐associated efflux, thereby activating antitumor immunity through P2X7R on immune cells. Chemiluminescence assay confirmed that PANX2‐OE enhanced eATP secretion, while PANX2‐KO reduced it (Figure 6D and Figure S6J). Co‐culture experiments demonstrated that PANX2‐OE A549 cells released increased eATP, which was associated with upregulated P2X7R expression on co‐cultured immune cells, thereby promoting their activation and recruitment. Conversely, PANX2‐KO reduced eATP levels, downregulated P2X7R, and consequently impaired immune cell signaling (Figure 6E,F).

To confirm the functional necessity of this pathway, we performed intervention experiments in the co‐culture system. As expected, the ATP‐hydrolyzing enzyme Apyrase, but not the P2X7R antagonist A438079, abrogated the PANX2‐OE‐induced elevation of eATP levels, confirming that P2X7R acts downstream of eATP (Figure 6G) [62, 63]. Both Apyrase and A438079 reversed the upregulation of P2X7R on immune cells induced by PANX2‐OE in A549 cells and, consequently, blunted the increase in key antitumor cytokines (Figure 6H,I). Notably, an equimolar concentration of A438079 had no significant effect on A549 cell viability (Figure S6K), excluding nonspecific tumor cell toxicity. Collectively, these results demonstrate that PANX2‐OE in LUAD cells activates antitumor immunity primarily by enhancing eATP release and engaging the P2X7R signaling pathway on immune cells.

### 2.7 PANX2 Overexpression Suppresses Tumor Growth In Vivo Through Disulfidptosis and Antitumor Immunity

To validate the tumor‐suppressive function of PANX2 and the causal involvement of the identified pathways in vivo, we established subcutaneous LUAD xenograft models in BALB/c nu/nu mice. Mice were randomly assigned to eight groups (*n* = 5 per group) receiving injections of genetically modified A549 cells: control (for OE), PANX2‐OE, control (for KO), PANX2‐KO, PANX2‐OE with NRF2‐KD, PANX2‐OE with NRF2‐KD and SLC7A11‐OE, PANX2‐OE with G6PD‐OE, or PANX2‐OE with intraperitoneal injection of the P2X7R antagonist A740003 (Figure S7A) [64, 65]. Two weeks after tumor cell inoculation, a humanized immune microenvironment was established by intravenous adoptive transfer of activated antigen‐specific hPBMCs, with reconstitution efficiency confirmed by flow cytometry (Figure S7B) [66, 67, 68, 69, 70, 71, 72].

**FIGURE 7:**
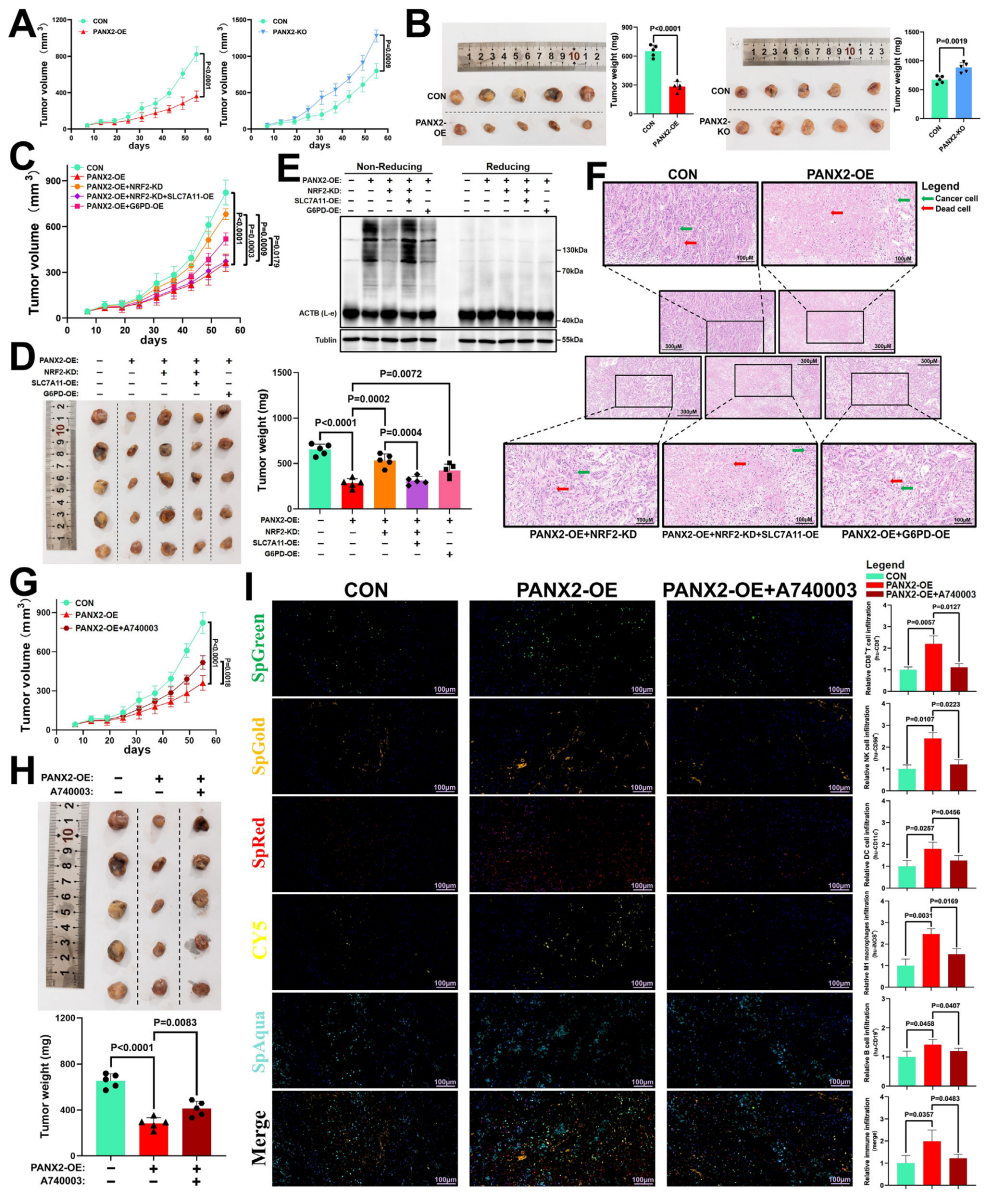
PANX2 overexpression suppresses tumor growth In vivo through disulfidptosis and antitumor immunity. (A) Tumor growth curves in PANX2‐OE/KO A549 xenografts vs. controls (*n* = 5 mice/group). (B) Gross tumor images and weights across PANX2‐OE/KO groups vs. controls (*n* = 5 mice/group). (C) Tumor growth curves to assess the effect of NRF2‐KD, NRF2‐KD+SLC7A11‐OE, G6PD‐OE on PANX2‐OE‐induced tumor suppression in xenografts. (D) Gross tumor images and weights in PANX2‐OE xenografts with the indicated protein expression interventions vs. PANX2‐OE alone. (E) Nonreducing SDS‐PAGE assay to assess disulfide crosslinking of cytoskeletal proteins in PANX2‐OE xenografts with the indicated protein expression interventions vs. PANX2‐OE alone and non‐PANX2‐OE controls. (F) H&E staining to assess cancer cell death in tumor tissues from PANX2‐OE xenografts with the indicated protein expression interventions vs. PANX2‐OE alone and non‐PANX2‐OE controls. (G) Tumor growth curves to assess the effect of A740003 treatment on PANX2‐OE‐induced tumor suppression in xenografts. (H) Gross tumor images and weights in PANX2‐OE xenografts with A740003 treatment vs. PANX2‐OE alone. (I) mIF to visualize immune infiltration in tumor tissues from PANX2‐OE xenografts with A740003 treatment vs. PANX2‐OE alone and non‐PANX2‐OE controls. (Data are presented as means ± SD; *n* ≥ 3 independent experiments; “OE,” “KO,” “KD,” and “CON” denote overexpression, knockout, knockdown, and control, respectively).

Consistent with in vitro findings, PANX2‐OE potently suppressed tumor growth, whereas PANX2‐KO accelerated progression (Figure 7A,B). This antitumor effect was effectively reversed by NRF2‐KD or G6PD‐OE, confirming the functional necessity of both identified axes in vivo. Re‐expression of SLC7A11 on the NRF2‐KD background restored tumor suppression, supporting that NRF2 acts primarily through SLC7A11 (Figure 7C,D).

Mechanistically, PANX2‐OE tumors exhibited elevated NRF2 and SLC7A11, suppressed G6PD, cystine accumulation, and NADPH depletion, whereas PANX2‐KO tumors showed the opposite molecular profile (Figure S7C,D). Crucially, both NRF2‐KD and G6PD‐OE interventions effectively alleviated the cystine overload and NADPH deficiency induced by PANX2‐OE (Figure S7E–H). Correspondingly, non‐reducing WB confirmed disulfide cross‐linking of cytoskeletal proteins in PANX2‐OE tumors, which was alleviated by NRF2‐KD or G6PD‐OE (Figure 7E). Hematoxylin and eosin (HE) staining revealed extensive cell death in PANX2‐OE tumors, a phenotype that was reduced upon disruption of these pathways (Figure 7F and Figure S7I). Importantly, re‐expression of SLC7A11 in the NRF2‐KD background restored the disulfidptosis phenotype, confirming its role as the key effector downstream of NRF2.

We next assessed whether PANX2 modulates antitumor immunity in vivo. Treatment with A740003 partially reversed PANX2‐OE‐induced tumor growth suppression (Figure 7G,H). In parallel, hematoxylin and eosin (H&E) staining and multiplex immunofluorescence (mIF) revealed markedly enhanced infiltration of antitumor immune cells—including CD8^+^ T and NK cells, among others—in PANX2‐OE tumors, an effect that was reversed by A740003 treatment, demonstrating its dependence on eATP‐P2X7R signaling. In contrast, PANX2‐KO tumors exhibited reduced immune cell accumulation, correlating with accelerated tumor growth (Figure 7I and Figure S7J,K).

Collectively, these in vivo data establish that PANX2‐OE constrains LUAD progression through a coordinated mechanism involving the induction of disulfidptosis and the promotion of an antitumor immune response.

### 2.8 PANX2 Expression Correlates With Altered Drug Sensitivity Profiles in Lung Cancer

To explore the broader potential clinical implications of PANX2, we analyzed transcriptomic data from the GSE40275 lung cancer cohort. Samples were stratified into high‐ and low‐PANX2 expression groups, and drug sensitivity to 198 clinical/preclinical agents was predicted using the oncoPredict algorithm. Differential analysis identified the top 20 drugs with the most significant sensitivity differences between the groups (Figure S8A).

The analysis revealed that high‐PANX2 tumors exhibited increased predicted sensitivity to agents with known effects on cellular metabolism and cytoskeletal integrity (e.g., Elephantin, Oxaliplatin) [73, 74, 75]. Conversely, they showed reduced sensitivity to inhibitors of growth factor pathways such as EGFR/MAPK and PI3K/AKT (e.g., AZD3759, Alpelisib) [76, 77].

Collectively, these in silico findings link PANX2 expression to distinct drug sensitivity patterns, suggesting its potential utility as a marker for differential therapeutic responses.

## 3 Discussion

Disulfidptosis is a recently identified form of programmed cell death driven by NADPH depletion and cystine overload, leading to pathological disulfide crosslinking of actin cytoskeletal proteins and ultimately cytoskeletal collapse. Cancer cells, with their heightened reliance on SLC7A11‐mediated cystine uptake and NADPH‐dependent redox homeostasis, exhibit unique susceptibility to this death modality. However, the upstream regulators of disulfidptosis in LUAD—particularly the interplay between metabolic enzymes and membrane channels—remain poorly characterized. In this study, we identify PANX2 as a critical tumor suppressor in LUAD that orchestrates tumor regression through a coordinated dual mechanism: intrinsically triggering disulfidptosis in cancer cells via convergent metabolic pathways, while extrinsically activating antitumor immunity through eATP‐P2X7R signaling. Unlike PANX1, which has been implicated in the regulation of apoptosis, pyroptosis, and inflammatory responses, PANX2 appears to function as a tumor suppressor in LUAD through its unique ability to induce disulfidptosis and activate antitumor immunity. This functional divergence likely stems from differences in channel properties, subcellular localization, and tissue‐specific expression patterns [17, 18, 19].

Clinically, we demonstrate that PANX2 expression is progressively downregulated with advancing LUAD stage, and that low PANX2 levels correlate with poor patient survival across multiple independent cohorts and tissue microarray validation. These observations establish PANX2 as a potential prognostic biomarker and suggest that its loss may represent an adaptive mechanism by which LUAD cells evade both metabolic catastrophe and immune elimination. Given that PANX2 is downregulated in multiple malignancies, including glioma, its tumor‐suppressive function may extend beyond LUAD, though whether it engages similar mechanisms in those contexts remains to be determined [22, 23].

Mechanistically, we identify disulfidptosis as the primary cell death modality underlying PANX2‐mediated tumor suppression. PANX2‐OE LUAD cells underwent a form of death resistant to classical apoptosis, necroptosis, autophagy, and ferroptosis inhibitors, but specifically rescued by disulfide reductants. This death exhibited the hallmark features of disulfidptosis: NADPH depletion, cystine overload, and pathological disulfide crosslinking of cytoskeletal proteins (ACTB, FLNA/B) leading to actin cytoskeletal collapse and actin‐plasma membrane detachment (Figure 3C). Importantly, we delineate two convergent metabolic axes through which PANX2 orchestrates this lethal cascade. First, PANX2 activates NRF2 through a Ca^2^^+^‐dependent mechanism that concurrently enhances both its protein stability and nuclear translocation, leading to transcriptional upregulation of the cystine transporter SLC7A11. This results in enhanced cystine influx and elevated intracellular cystine levels, which in turn increases NADPH consumption for cystine reduction. Second, PANX2 suppresses G6PD, the rate‐limiting enzyme of the PPP, thereby impairing NADPH regeneration and compromising the clearance of accumulated cystine. The synergy between these two pathways is essential, as PANX2 simultaneously drives cystine influx while crippling the NADPH supply needed for its reduction, creating a “perfect storm” of NADPH depletion and cystine overload. This dual regulation triggers pathological disulfide crosslinking of actin cytoskeletal proteins, culminating in actin cytoskeletal collapse and disulfidptosis. Rescue experiments—including NRF2‐KD, SLC7A11‐KD, G6PD‐OE, and re‐expression of SLC7A11 in the NRF2‐KD background—confirmed that both axes are necessary and act synergistically. Notably, PANX2‐induced disulfidptosis exhibited LUAD specificity, with minimal effects on normal lung epithelial cells, likely reflecting the inherently high metabolic demand and redox vulnerability of cancer cells. While our study focuses on these two core axes, the thioredoxin system may also contribute, as evidenced by TRi‐1 exacerbating PANX2‐induced cell death. Future studies should explore potential crosstalk with other NADPH‐generating pathways, NADPH‐consuming pathways, and additional antioxidant systems. Given that recent work has classified lung cancer among the cancer types with the highest disulfidptosis susceptibility, it will be important to investigate whether the tumor‐suppressive effect of PANX2 is restricted to intrinsically sensitive lineages or can be extended to less susceptible cancers [78].

Beyond this cell‐intrinsic death mechanism, we uncover a previously unknown immunomodulatory role for PANX2. Using an A549‐hPBMC co‐culture system with detailed flow cytometry immunophenotyping, we found that PANX2‐OE significantly enhanced the infiltration and activation of multiple immune subsets—including CD8^+^ T cells, cytotoxic CD8^+^CD107a^+^ subsets, NK cells (with polarization toward the cytotoxic CD56dimCD16^+^ population), DC cells, M1 macrophages, and B cells—effectively remodeling the tumor immune microenvironment into an immunologically “hot” state. This was accompanied by elevated levels of antitumor cytokines and chemokines (IFN‐γ, IL‐12, CXCL9, CXCL10) and reduced immunosuppressive factors (IL‐6, CCL2), as measured by multiplex ELISA. Mechanistically, we link this effect to PANX2's canonical function as an ATP‐permeable channel. PANX2‐OE A549 cells released elevated levels of eATP, which acts as a potent DAMP signaling through the P2X7 receptor on immune cells. This was confirmed by P2X7R blockade or ATP degradation, which reversed immune activation and infiltration both in the co‐culture system and in vivo. This finding directly connects a metabolic cell death pathway (disulfidptosis) with immunogenic signaling, suggesting a self‐amplifying loop where PANX2‐induced cell death further augments immune activation through enhanced ATP release. Beyond ATP, PANX2 overexpression also altered the secretion of multiple chemokines and cytokines, suggesting broader immunomodulatory effects that may shape the immune microenvironment through direct channel substrates or indirect consequences of immune cell activation.

The in vivo relevance of these findings was validated in a humanized mouse model, where PANX2‐OE significantly suppressed tumor growth, concomitant with increased cystine accumulation, NADPH depletion, disulfide crosslinking, and enhanced antitumor immune infiltration, confirmed by multiplex immunofluorescence staining for CD8^+^ T cells, NK cells, DC cells, and M1 macrophages (Figure 7I and Figure S7K), leading to extensive tumor necrosis. Notably, these effects were attenuated by NRF2‐KD, G6PD‐OE, or P2X7R blockade, confirming the essential contribution of both pathways. Furthermore, our drug sensitivity analysis predicted that high‐PANX2 LUAD tumors exhibit increased sensitivity to agents targeting cellular metabolism and cytoskeletal integrity, while showing reduced sensitivity to inhibitors of growth factor pathways such as EGFR/MAPK and PI3K/AKT. This suggests that PANX2 expression may serve as a predictive biomarker for personalized therapy, guiding the selection of metabolic inhibitors or immunotherapeutic strategies for LUAD patients.

Our study has several limitations that warrant further investigation. First, the precise upstream signals regulating PANX2 expression in LUAD remain unknown. Second, while we demonstrate that PANX2 regulates NRF2 through a Ca^2^^+^‐dependent mechanism involving both protein stabilization and nuclear translocation, the detailed molecular link between PANX2 channel activity and NRF2 regulation requires further dissection. Third, the mechanism by which PANX2 suppresses G6PD requires additional investigation. Fourth, although we focused on the actin cytoskeleton as the primary target of disulfide stress, whether PANX2‐induced disulfidptosis affects other cytoskeletal components remains to be explored. Finally, while our xenograft model with immune reconstitution provides compelling evidence, future studies using patient‐derived xenografts or genetically engineered mouse models would better recapitulate clinical LUAD heterogeneity.

In conclusion, this study establishes PANX2 as a master regulator that constrains LUAD progression by concurrently activating a tumor‐selective metabolic death pathway (disulfidptosis) and reprogramming the tumor microenvironment toward immune effectorism. Our findings not only expand the understanding of disulfidptosis regulation but also reveal a novel, therapeutically appealing strategy to simultaneously target cancer cell metabolism and antitumor immunity.

## 4 Experimental Section

### 4.1 Analysis of Clinical Data and Patient Prognosis

Transcriptomic data were obtained from public databases for differential expression and survival analysis. The GEO dataset GSE40275, containing 43 normal lung tissues and 37 primary lung cancer tissues (excluding 4 metastatic cases), was used to analyze PANX2 expression across normal vs. tumor tissue and different clinical stages (Stage I vs. II‐III) (Table S1). For LUAD‐specific prognostic validation, data from The Cancer Genome Atlas (TCGA; *n* = 490 LUAD patients; Table S2) and the Human Protein Atlas (HPA; *n* = 173 LUAD patients; Table S3) were used. Patients were stratified into high‐ and low‐PANX2 expression groups based on an optimal cut‐off value (maxstat R package). Kaplan‐Meier survival analyses for overall survival (OS), disease‐free interval (DFI), and progression‐free interval (PFI) were performed, and curves were compared using the log‐rank test. Hazard ratios (HR) and 95% confidence intervals (CI) were calculated with the survival R package.

### 4.2 Tissue Microarray and Immunohistochemistry

PANX2 protein expression was quantified by immunohistochemistry using two LUAD‐specific tissue microarray ((TMA; OUTDO Biotech, XT24‐015) containing 60 paired tumor and adjacent normal tissues, stratified by clinical stage (Stage I: *n* = 12; Stage II: *n* = 28; Stage III: *n* = 20). TMA sections were dewaxed, subjected to antigen retrieval in 10 mM EDTA buffer (pH 9.0) at 95°C for 20 min, and endogenous peroxidase activity was blocked with 3% H_2_O_2_ (Sigma, H1009). Sections were incubated with anti‐PANX2 rabbit polyclonal antibody (1:200; Proteintech, 26604‐1‐AP) at 37°C for 90 min, followed by a universal mouse/rabbit secondary antibody (ABclonal, AS014). Staining was developed with 3,3'‐diaminobenzidine (DAB; Beyotime, P0202) and counterstained with hematoxylin (Solarbio, IH0030). Staining intensity was quantitatively scored using ImageJ software.

### 4.3 Cell Culture and Genetic Engineering

#### 4.3.1 Cell Lines and Culture Conditions

Human LUAD cell lines, including A549 (TCHu150; obtained in 2021), NCI‐H1299 (TCHu160; 2022), NCI‐H358 (TCHu151; 2023), PC‐9 (SCSP‐5085; 2023), NCI‐H1650 (TCHu152; 2023), NCI‐H1975 (TCHu193; 2024), Calu‐3 (TCHu157; 2024), NCI‐H292 (TCHu122; 2023), as well as normal bronchial epithelial cell line BEAS‐2B (GNHu27; 2023), were obtained from the Cell Bank of the Chinese Academy of Sciences (Shanghai, China). The human bronchial epithelial cell line 16HBE, originally established by Cozens et al. [79], was obtained from XYBiotechnology (Shanghai, China; XY‐H444; 2022). All cell lines were STR‐profiled and routinely confirmed mycoplasma‐free. Cells were cultured in DMEM (Gibco, 11965092) supplemented with 10% FBS (Gibco, 10099141) and 1% penicillin‐streptomycin (Gibco, 15140122) at 37°C with 5% CO_2_. Human peripheral blood mononuclear cells (hPBMCs) from healthy donors were purchased from Meisen Cell Technology (Zhejiang, China) and cultured in F‐12 medium (Gibco, 11330032). For cystine starvation treatment, cells were incubated in customized cystine‐free medium (Gibco) for the indicated time before sample preparation.

#### 4.3.2 Lentivirus Production and Transduction

Lentiviruses for all stable genetic manipulations were produced in 293T cells. Cells were co‐transfected with the gene transfer plasmid, the packaging plasmid psPAX2 (Addgene, 12260), and the envelope plasmid pVSVg (Addgene, 8454) using Lipofectamine 3000 (Invitrogen, L3000008). Viral supernatants were collected at 48–72 h, filtered (0.45 µm), and concentrated by ultracentrifugation. Target cells (A549, H1299, BEAS‐2B) were transduced in the presence of polybrene (8 µg/mL, Solarbio, IR9121). Stable polyclonal populations were selected with appropriate antibiotics for 10–14 days, as detailed below for each specific genetic model.

#### 4.3.3 PANX2 Overexpression and Knockout Models

For stable PANX2 overexpression (PANX2‐OE), the human PANX2 cDNA was cloned into the pLVX‐FLAG‐Puro vector (MiaoLingBio), verified by sequencing, and packaged into lentivirus. Control cells were transduced with the empty vector. Both were selected with puromycin (2 µg/mL; Solarbio, IP12803). For CRISPR/Cas9‐mediated knockout (PANX2‐KO), target cells were first transduced with a lentiviral vector expressing Cas9 (pCDH‐CAG‐Cas9‐T2A‐HygR, VectorBuilder) and selected with hygromycin B (100 µg/mL; Sigma, H3274). Subsequently, these Cas9‐expressing cells were transduced with a lentivirus expressing a PANX2‐targeting sgRNA (cloned into a puromycin‐resistant vector). Control cells received a non‐targeting control sgRNA. Double‐positive cells were selected with both hygromycin B and puromycin.

#### 4.3.4 Knockdown and Rescue Models

For stable knockdown, shRNA constructs targeting NRF2 or SLC7A11 were cloned into the pLKO.1‐Hygro vector (MiaoLingBio) and transduced into PANX2‐OE cells. Controls received a nontargeting shRNA. Double‐modified pools were selected with hygromycin B. For rescue experiments, cDNAs of G6PD or SLC7A11 were cloned into vectors with distinct resistance markers (e.g., blasticidin S). These were transduced into PANX2‐OE cells (for G6PD‐OE) or PANX2‐OE+NRF2‐KD cells (for SLC7A11‐OE), followed by selection with the corresponding antibiotic (blasticidin S; 10 µg/mL; Solarbio, B9300).

### 4.4 Molecular Biology Assays

#### 4.4.1 Western Blot (WB) Analysis

Proteins were extracted from cultured cells or snap‐frozen tumor tissues using RIPA Lysis Buffer (Beyotime, P0013B) supplemented with protease inhibitors. For tissue samples, tumors were homogenized in RIPA buffer using a tissue homogenizer, followed by centrifugation at 12 000 × g for 15 min at 4°C to collect the supernatant. For analysis under non‐reducing conditions, lysates were treated with 20 mM N‐ethylmaleimide (NEM; Sigma, E3876). Protein concentration was determined using a BCA assay. Equal amounts of protein were separated by SDS‐PAGE and transferred onto PVDF membranes. After blocking, membranes were incubated overnight at 4°C with specific primary antibodies (listed in Table S4). Following washes, membranes were incubated with HRP‐conjugated secondary antibodies (CST, 7074S/7076S). Protein bands were visualized using enhanced chemiluminescence (ECL) substrate (Meilunbio, MA0186‐2) and quantified using ImageJ software.

#### 4.4.2 Quantitative Real‐Time PCR (qRT‐PCR)

Total RNA was isolated from cells or tissues using TRIzol reagent (Invitrogen, 15596026). For tissue samples, tumors were homogenized in TRIzol reagent using a tissue homogenizer before proceeding with RNA extraction. RNA concentration and purity were assessed. One microgram of total RNA was reverse‐transcribed into cDNA using the HiScript III Reverse Transcriptase Kit (Vazyme, R323‐01). Quantitative real‐time PCR was performed in triplicate using PowerUp SYBR Green Master Mix (Thermo Fisher Scientific, A25742) on an Applied Biosystems 7500 system. The thermal cycling conditions were: 95°C for 2 min, followed by 40 cycles of 95°C for 15 s and 60°C for 1 min, with a final melt curve analysis. Gene expression was normalized to GAPDH and calculated using the 2−ΔΔCt method. Primer sequences are provided in Table S5.

#### 4.4.3 Protein Stability and Subcellular Fractionation

To assess NRF2 protein stability, cells were treated with 40 µg/mL cycloheximide (CHX; Sigma, C7698) and harvested at the indicated time points for WB analysis. For subcellular fractionation, nuclear and cytoplasmic proteins were separated using the NE‐PER Kit (Thermo Fisher Scientific, 78833). NRF2 protein levels in each fraction were analyzed by WB, with Lamin B1 and GAPDH serving as compartment‐specific loading controls.

#### 4.4.4 Ca^2^^+^ Flux Measurement

Intracellular Ca^2^^+^ levels were assessed using Fluo‐4 AM (Beyotime, S1060). Cells seeded in 96‐well plates were loaded with 5 µM Fluo‐4 AM at 37°C for 30 min, washed, and then incubated in dye‐free buffer. For Ca^2^^+^ chelation experiments, LUAD cells were preincubated with 10 µM BAPTA‐AM (Aladdin, B421097) for 30 min prior to Fluo‐4 AM loading. Fluorescence intensity (Ex/Em = 488/516 nM) was measured using a microplate reader (Thermo Fisher Scientific) and normalized to cell number.

### 4.5 Assessment of Disulfidptosis and Cellular Metabolism

#### 4.5.1 Cystine Uptake Assay

Cystine uptake was measured using a commercial fluorometric assay kit (Elabscience, E‐BC‐F066) according to the manufacturer's instructions. Briefly, harvested cells were incubated with a pre‐warmed cystine analog working solution or PBS (control) at 37°C for 30 min. After ethanol treatment and centrifugation, the supernatants were incubated with the assay working solution at 37°C for 30 min. Fluorescence intensity (Ex/Em = 485/535 nM) was then measured using a microplate reader (Thermo Fisher Scientific). Net fluorescence (assay minus control) was calculated and normalized based on the initial cell number.

#### 4.5.2 Cystine Level Detection

Cystine levels in cultured cells and tumor tissues were quantified using a human Cystine ELISA Kit (Bioesn, BES0473K). Cells were lysed in PBS. Tumor tissues were excised, rinsed thoroughly with ice‐cold PBS to remove residual blood, and homogenized in PBS. The lysates and homogenates were cleared by centrifugation, and supernatants were collected. Samples and cystine standards were added to precoated wells and incubated at 37°C for 60 min. After washing, a biotinylated detection antibody was added, followed by incubation and washing. Streptavidin‐biotin‐peroxidase complex (SABC) was then added. Following a final wash, the reaction was developed with TMB substrate, stopped, and the absorbance was measured at 450 nM. Cystine concentration was determined from the standard curve and normalized to total protein content determined by a BCA assay.

#### 4.5.3 Reactive Oxygen Species (ROS) Detection

Intracellular ROS levels were measured using a ROS Assay Kit (Beyotime, S0033S). Briefly, cells were loaded with 10 µM DCFH‐DA (diluted in serum‐free medium) and incubated at 37°C for 20 min. After washing with PBS to remove excess probe, the cells were treated as required. Fluorescence intensity (Ex/Em = 488/525 nM) was measured using a microplate reader and expressed as relative fluorescence units (RFU).

#### 4.5.4 NADP^+^/NADPH Ratio Assay

The NADP^+^/NADPH ratio in cultured cells and tumor tissues was determined using a colorimetric assay kit (Elabscience, E‐BC‐K803‐M). Cells were lysed, and tissues were homogenized in the provided extraction buffer. The lysates or homogenates were centrifuged, and the supernatants were filtered through a 10‐kDa cutoff filter to remove NADPH‐degrading enzymes. To measure total NADP(H), the filtered lysate was used directly. To specifically measure NADPH, an aliquot of the filtered lysate was heated at 60°C for 30 min to decompose NADP^+^. For the assay, 50 µL of each sample was incubated with 100 µL of reaction working solution at 37°C for 10 min, followed by addition of 20 µL of chromogenic agent and a further 10‐min incubation. Absorbance at 450 nM was measured using a microplate reader. Concentrations were calculated based on an NADPH standard curve, and the NADP^+^/NADPH ratio was derived and normalized to the total protein content determined by a BCA assay.

#### 4.5.5 G6PD Activity Assay

Glucose‐6‐phosphate dehydrogenase (G6PD) activity was measured using a colorimetric assay kit (Elabscience, E‐BC‐K056‐M). Cell lysates were prepared in the provided extraction buffer. Sample aliquots were assayed in parallel test and control wells using the respective reaction working solutions. After incubation at 37°C for 10 min, absorbance at 450 nM was measured using a Thermo Fisher Scientific microplate reader.

#### 4.5.6 F‐Actin and Nuclear Staining

F‐actin and nuclei were visualized by co‐staining with Alexa Fluor 594‐conjugated phalloidin (Cell Navigator F‐Actin Labeling Kit; AAT Bioquest, 22664) and DAPI (Beyotime, C1005), respectively. Cells on coverslips were fixed, permeabilized, and stained according to the manufacturer's protocols. Images were acquired on a Zeiss LSM 880 confocal microscope (F‐actin: Ex/Em = 594/610 nM; DAPI: Ex/Em = 364/454 nM) and analyzed using ZEN software.

#### 4.5.7 F‐Actin and Plasma Membrane Co‐staining

For simultaneous visualization of F‐actin and the plasma membrane, cells were first fixed, permeabilized, and stained for F‐actin with Alexa Fluor 594‐phalloidin. Subsequently, the plasma membrane was labeled using the green fluorescent dye DiO (Cell Plasma Membrane Staining Kit; Beyotime, C1993S) according to the manufacturer's instructions. Images were acquired on a Zeiss LSM 880 confocal microscope using the corresponding green (DiO, Ex/Em = 484/501 nM) and red (phalloidin) fluorescence channels and analyzed for co‐localization.

#### 4.5.8 Intracellular ATP Detection

Intracellular ATP levels were measured using the Enhanced ATP Assay Kit (Beyotime, S0027). Cells were lysed in the provided lysis buffer, and the supernatant was collected after centrifugation. The assay working solution was prepared by diluting the detection reagent 1:4. For detection, 100 µL of working solution and 20 µL of sample or ATP standard were combined in a well, and luminescence was measured immediately using a Varioskan LUX microplate reader. ATP concentration was calculated from a standard curve (0.01–10 µM) and normalized to the total protein content.

#### 4.5.9 Glucose Uptake Assay

Glucose uptake was assessed using the Glucose Uptake Assay Kit (Dojindo, UP02). Cells were starved for 2 h before incubation with 2‐NBDG (100 µM) for 30 min. Fluorescence intensity (Ex/Em = 480/520 nM) was measured using a Thermo Fisher Scientific microplate reader.

#### 4.5.10 Cell Viability Assay

Cell viability was assessed using the Cell Counting Kit‐8 (CCK‐8; GOONIE, 100–106). Cells were seeded in 96‐well plates at a density of 1 × 10^4^ cells per well. After 24 h, cells were subjected to the indicated genetic or pharmacological treatments. Following treatment, 10 µL of CCK‐8 reagent was added to each well and incubated at 37°C for 1 h. Absorbance was measured at 450 nM using a Thermo Fisher Scientific microplate reader.

#### 4.5.11 Cell Death Modality and Pathway Validation Assay

To investigate the specific death modality, PANX2‐OE LUAD cells were incubated with various inhibitors for 24 h. The inhibitors used included: Z‐VAD‐FMK (10 or 20 µM; AbMole, M3143), necrostatin‐1 (Nec‐1; 5 or 10 µM; AbMole, M2315), chloroquine (CQ; 20 or 40 µM; AbMole, M9559), AC‐YVAD‐CMK (10 or 20 µM; AbMole, M8340), ferrostatin‐1 (Fer‐1; 5 or 10 µM; AbMole, M2698), N‐acetylcysteine (NAC; 5 mM; Aladdin, A105420), dithiothreitol (DTT; 2 mM; AbMole, M9093), tris(2‐chloroethyl) phosphate (TCEP; 1 mM; AbMole, M55340), PX‐12 (10 µM; AbMole, M7929), and TRi‐1 (10 µM; AbMole, T422841). Cell viability was then determined by CCK‐8 assay. The efficacy of each inhibitor was validated in parallel positive‐control experiments using the following death inducers: staurosporine (STS; 0.5 µM; AbMole, M2066) for apoptosis, RIP1/RIP3/MLKL activator 1 (2 µM; Aladdin, R651898) for necroptosis, rapamycin (100 nM; AbMole, M1768) for autophagy, nigericin (20 µM; AbMole, M9780; with LPS priming) for pyroptosis, and RSL3 (1 µM; AbMole, M9060) for ferroptosis. To further validate the role of SLC7A11, NRF2, and G6PD, PANX2‐OE cells were treated with sulfasalazine (SAS; 2 mM), ML385 (10 µM), or AG1 (5 µM) for 24 h, alone or in combination. Cell viability was then determined by CCK‐8 assay.

#### 4.5.12 Cell Death Detection

Cell death was quantified by flow cytometry using the Zombie NIR Fixable Viability Dye (BioLegend, 423107). Cells were stained with the dye (1:1000 dilution) for 20 min at 37°C, washed, and analyzed on a Cytek Aurora flow cytometer. Data were processed using FlowJo v10 software to determine the percentage of dead cells.

### 4.6 Malignant Phenotype Assays

#### 4.6.1 Cell Proliferation Assay

Cell proliferation was measured using the CCK‐8 kit (GOONIE, 100–106). Cells were seeded in 96‐well plates at a density of 3 × 10^3^ cells per well. At 0, 24, 48, and 72 h postseeding, 10 µL of CCK‐8 reagent was added to each well, followed by incubation at 37°C for 1 h. Absorbance was measured at 450 nM using a Thermo Fisher Scientific microplate reader. Proliferation curves were generated by plotting absorbance values against time.

#### 4.6.2 Colony Formation Assay

For colony formation, 300 cells were seeded per well in 6‐well plates and cultured for 14 days. Colonies were fixed with 4% paraformaldehyde, stained with 0.1% crystal violet, and imaged. Colonies consisting of ≥50 cells were counted under a microscope; quantitative analysis was performed using ImageJ software.

#### 4.6.3 Scratch Assay

Scratch wounds were created with a 200‐µL pipette tip. Cells were washed with PBS and incubated in serum‐free medium. Images were captured at 0 and 24 h. Wound healing rate = (initial width—final width)/initial width.

#### 4.6.4 Transwell Migration Assay

For migration, 3 × 10^4^ cells suspended in serum‐free medium were seeded into the upper chamber of Transwell inserts (8 µm pore; Corning, 353097). After 24 h incubation, cells on the lower membrane were fixed with methanol, stained with 0.1% crystal violet, and imaged. Migrated cells were counted in five random fields per insert using ImageJ software.

#### 4.6.5 Transwell Invasion Assay

For invasion, Transwell inserts were precoated with Matrigel. Cells (3 × 10^4^) in serum‐free medium were seeded into the upper chamber and incubated for 24 h. Invaded cells on the lower membrane were fixed, stained, and counted as described for the migration assay.

### 4.7 Immune Modulation Assays

#### 4.7.1 Cancer–Immune Cell Co‐culture

A549 cells (PANX2‐OE, PANX2‐KO, and respective controls) were co‐cultured with hPBMCs at a 1:5 ratio in F12 medium + 10% FBS. After 72 h, the characteristics of immune cells were observed and photographed under a microscope. Subsequently, the culture supernatant was collected to detect the contents of cytokines and chemokines, and all cells were collected for flow cytometric immunophenotyping.

#### 4.7.2 Cytokine and Chemokine Detection

Levels of cytokines and chemokines (IFN‐γ, IL‐12, TNF‐α, CXCL9, CXCL10, IL‐2, IL‐6, CCL2) in co‐culture supernatants were quantified using commercial ELISA kits (JSBOSSEN). Absorbance at 450 nM was measured using a Sunrise F50 microplate reader (Tecan), and concentrations were derived from standard curves.

#### 4.7.3 Flow Cytometric Immunophenotyping

Co‐cultured cells were collected and aliquoted for multi‐color staining. Cell suspensions were incubated with fluorescently conjugated monoclonal antibodies against CD3, CD4, CD8, CD56, CD11c, CD14, CD86, CD206, and CD19 for 20–30 min at 37°C in the dark. After washing with PBS to remove unbound antibodies, cells were resuspended and analyzed on a Cytek Aurora flow cytometer. Instrument voltages and compensation were adjusted using single‐stained controls. Data from at least 10 000 events per sample were collected and analyzed using FlowJo v10 software to determine the proportions of distinct immune cell subsets; the gating strategy is shown in Figure S9A,B.

#### 4.7.4 Extracellular ATP (eATP) Measurement

eATP in supernatants from monocultures or co‐cultures was measured using the Enhanced ATP Assay Kit (Beyotime, S0027). Luminescence was recorded on a Thermo Fisher Varioskan LUX multimode microplate reader, and concentrations were calculated from a standard curve.

#### 4.7.5 P2X7R Signaling Blockade

To inhibit the ATP–P2X7R signaling, co‐cultures were treated with the P2X7R antagonist A438079 (10 µM; AbMole, M11483) or the ATP‐hydrolyzing enzyme Apyrase (10 U/mL; MCE, HY‐P2764) for 72 h. A control group received heat‐inactivated Apyrase (95°C for 15 min) under identical conditions.

### 4.8 In Vivo Studies

#### 4.8.1 Animal Ethics and Subcutaneous Tumor Model

All animal experiments were approved by the Institutional Animal Ethics Committee (Approval No. HWT‐BG‐117b, 202411003) and conducted in accordance with the ARRIVE guidelines. Male BALB/c nu/nu mice (4–6 weeks old, 16–18 g, γ‐irradiated) were acclimatized for one week under specific pathogen‐free conditions. Mice were randomly divided into 8 groups (*n* = 5 per group) and subcutaneously injected into the right axilla with 1 × 10^6^ A549 cells (suspended in 100 µL of saline) stably engineered as follows: Control‐OE, PANX2‐OE, Control‐KO, PANX2‐KO, PANX2‐OE+NRF2‐KD, PANX2‐OE+NRF2‐KD+SLC7A11‐OE, PANX2‐OE+G6PD‐OE, PANX2‐OE with intraperitoneal administration of A740003 (10 mg/kg every day starting on the day after hPBMC transfer).

#### 4.8.2 Human Immune Reconstruction and Tumor Monitoring

When the average tumor volume reached approximately 80 mM^3^ (on day 14 postinoculation), hPBMCs were adoptively transferred to reconstitute the human immune system in vivo. Briefly, hPBMCs were preactivated ex vivo with 20 ng/mL IL‐2 (PeproTech, 200–02) and 20 ng/mL IL‐15 (PeproTech, 200–15). To provide antigen‐specific stimulation, inactivated A549 cell lysate (prepared by three rapid freeze‐thaw cycles) was added to the culture. After 72 h of activation, 5 × 10^6^ cells per mouse in 100 µL PBS were injected intravenously via the tail vein. One week after hPBMC transfer, one mouse from each group was randomly selected, and peripheral blood was collected for flow cytometric analysis of human CD45^+^ cell percentages to confirm successful immune reconstitution (40%–60%). Tumor dimensions (length and width) were measured every 3 days using a digital caliper, and volume was calculated using the formula: V = 0.5 × length × width^2^.

#### 4.8.3 Tissue Harvest and Processing

Mice were euthanized by CO_2_ asphyxiation when the tumor volume in the control group approached ∼800 mM^3^. Tumors were carefully excised, weighed, and divided for subsequent analyses. One portion was snap‐frozen in liquid nitrogen and stored at ‐80°C for protein and RNA extraction. Another portion was fixed in 4% paraformaldehyde (PFA; Beyotime, P0099) for 24 h at 4°C, followed by paraffin embedding for histological examination.

#### 4.8.4 Hematoxylin and Eosin (H&E) Staining

Formalin‐fixed, paraffin‐embedded (FFPE) tumor tissues were sectioned at 5 µm thickness. Sections were deparaffinized in xylene and rehydrated through graded ethanol. Staining was performed using a Hematoxylin and Eosin (H&E) Staining Kit (Solarbio, G1120) following the manufacturer's instructions, including hematoxylin staining, differentiation, bluing, and eosin counterstaining. Finally, sections were dehydrated, cleared in xylene, mounted with neutral balsam, and imaged using a light microscope (Nikon Eclipse Ci‐L).

#### 4.8.5 Multiplex Immunofluorescence (mIF) Staining

For immune phenotyping, sequential FFPE sections were stained using an Opal 7‐Color Manual IHC Kit (Akoya, NEL821001KT). After antigen retrieval (EDTA buffer, pH 9.0), sections were sequentially incubated with primary antibodies against CD8 (1:200; CST, 85336S), CD56 (1:200; Abcam, ab75813), CD11c (1:200; CST, 97585S), iNOS (1:200; Abcam, ab178945), CD163 (1:200; Abcam, ab182422), and CD19 (1:200; CST, 90176S). Each primary antibody was followed by an HRP‐conjugated secondary antibody and tyramide signal amplification (TSA) with a distinct Opal fluorophore. Nuclei were counterstained with DAPI (Beyotime, C1005). Multispectral images were acquired on a Vectra Polaris system (Akoya) and analyzed using inForm software (Akoya) to quantify immune cell density and distribution. The color scheme is shown in Figure S9C,D.

### 4.9 Bioinformatic Analysis

#### 4.9.1 Gene Set Enrichment Analysis (GSEA)

GSEA was performed on the primary lung cancer transcriptomic data from the GSE40275 dataset. Using the clusterProfiler R package, samples were stratified into high‐ and low‐PANX2 expression groups based on a tertile split (middle tertile excluded). Enrichment was assessed against gene sets from the Molecular Signatures Database (MSigDB), covering Gene Ontology (GO) biological processes, molecular functions, and Kyoto Encyclopedia of Genes and Genomes (KEGG) pathways. Parameters were set to: minimum size = 15, maximum size = 500, permutations = 1000 (Table S6). Enriched terms were ranked by enrichment score, and the top significant terms were selected for visualization.

#### 4.9.2 Gene Expression Correlation Analysis

Using the same transcriptomic data, pairwise Pearson correlation coefficients between target genes were calculated (Table S7). Statistical significance was defined as *p* < 0.05, and relationships were visualized using scatter plots.

#### 4.9.3 Single‐Sample GSEA (ssGSEA) of Immune Cells

ssGSEA was performed on the same transcriptomic data using the GSVA R package. Immune cell signature gene sets were obtained from the ImmPort database. Enrichment scores were calculated for each sample to quantify immune cell infiltration, and these scores were compared between the same high‐ and low‐PANX2 expression groups (Table S8).

#### 4.9.4 Drug Sensitivity Prediction

Drug sensitivity scores (derived IC50 values, converted from original IC50) for the same transcriptomic data were predicted for 198 clinical and preclinical compounds using the oncoPredict R package, with the pretrained GDSC2 model as reference. Samples were stratified into the same high‐ and low‐PANX2 expression groups. Differential sensitivity between groups was analyzed using the limma package (Table S9). Drugs were ranked by significance, and the top candidates were selected for visualization.

### 4.10 Statistical Analysis

Data are presented as mean ± SD from ≥3 independent experiments. For comparisons between two groups, Student's unpaired *t*‐test or Mann–Whitney U test was used as appropriate. For multiple groups, one‐way or two‐way ANOVA (with Tukey's post hoc test) or Kruskal–Wallis test (with Dunn's post hoc test) was applied. All analyses were performed in GraphPad Prism 9.0, with *p* < 0.05 considered statistically significant.

## Author Contributions

Y.C. performed conceptualization, methodology, software, validation, investigation, data curation, visualization, writing – original draft, writing – review & editing, project administration, manuscript submission, and handled correspondence during peer review. Z.‐Y.L. performed formal analysis, supervision, and data interpretation. G.‐W.Q. and R.‐H.Z. conducted validation. W.‐X.Y and Y.‐B.Z. provided resources. X.L. was responsible for project administration, supervision, and funding acquisition. Y.‐X.L. was responsible for conceptualization, formal analysis, project administration, writing – review & editing, and supervision.

## Conflicts of Interest

The authors declare no conflicts of interest.

## Supporting information




**Supporting File 1**: advs75662‐sup‐0001‐SuppMat.docx.

**Supporting File 2**: advs75662‐sup‐0002‐FiguresS1‐S9.zip.

**Supporting File 3**: advs75662‐sup‐0003‐TableS1‐S9.zip.

## Data Availability

The data that support the findings of this study are available from the corresponding author upon reasonable request.
