# Supplementary material for: PANX2 Suppresses Lung Adenocarcinoma Progression by Inducing Disulfidptosis and Enhancing Antitumor Immunity
Source: Adv Sci (Weinh). 2026 May 29:e75662. Online ahead of print. doi: 10.1002/advs.75662 (PMC13335910; doi:10.1002/advs.75662)
Supplement: Supplementary file 1 — Supporting File 1: advs75662‐sup‐0001‐SuppMat.docx. [file ADVS-9999-e75662-s003.docx]

**Supporting Information**

PANX2 Suppresses Lung Adenocarcinoma Progression by Inducing Disulfidptosis and Enhancing Antitumor Immunity

Yi Chen*, Zhao-Yu Liu, Gao-Wen Qu, Run-Hao Zeng, Wen-Xia Yao, Yong-Bing Zhou, Xue Liang*, and Yu-Xiong Lai*

**This PDF file includes:**

Figures S1 to S9

Tables S1 to S9

Figure S1.

**Figure S1.** A) qRT-PCR quantification of PANX2 mRNA levels in diverse cell lines. B) Validation of PANX2-OE/KO protein levels by WB, and mRNA levels by qRT-PCR. D) CCK-8 assay for proliferation in PANX2-OE/KO cells vs. controls. E) Colony formation assay for PANX2-OE/KO cells vs. controls. F) Scratch assay for migration capacity in PANX2-OE/KO cells vs. controls. G) Transwell migration assay in PANX2-OE/KO cells vs. controls. H) Transwell invasion assay to evaluate invasive phenotype.

Figure S2.

**Figure S2.** A) Microscopic observation of A549 cell morphology upon PANX2-OE/KO. B-D) GSEA of high/low-PANX2 lung cancer from GSE40275: GO BP (B), GO MF (C), KEGG (D). E) GSEA enrichment summary table. F) CCK-8 assay of inhibitor effects on PANX2-OE LUAD cells: Z-VAD-FMK (10/20 μM), Nec-1 (5/10 μM), CQ (20/40 μM), AC‑YVAD‑CMK (10/20 μM), Fer-1 (5/10 μM), 24 h. G) CCK-8 assay validating cell death inhibitors: Z-VAD-FMK for staurosporine (STS, 0.5 μM)-induced apoptosis, Nec-1 for RIP1/RIP3/MLKL activator 1 (R/R/M 1, 2 μM)-induced necroptosis, CQ for rapamycin (100 nM)-induced autophagy, AC-YVAD-CMK for nigericin (20 μM, LPS pretreatment)-induced pyroptosis, and Fer-1 for RSL3 (1 μM)-induced ferroptosis in A549 cells, 24 h. H) WB analysis of cell death executioner proteins in LUAD cells upon PANX2-OE: Cleaved Caspase-3 (apoptosis, STS as positive control), p-MLKL (necroptosis, R/R/M 1 as positive control), LC3B-I/II (autophagy, rapamycin as positive control), Cleaved GSDMD (pyroptosis, LPS priming followed by nigericin as positive control), and GPX4 (ferroptosis, RSL3 as positive control). I) Intracellular ATP detection: PANX2-OE does not deplete ATP levels in LUAD cells. J) ROS detection: PANX2-OE increases intracellular ROS levels in LUAD cells. K) CCK-8 assay: NAC fails to rescue PANX2-OE-induced cell death in LUAD cells.

Figure S3.

**Figure S3.** A) Non-reducing SDS-PAGE: PANX2-OE induces disulfide crosslinking of cytoskeletal proteins in H1299 cells and DTT (2 mM, 24 h) reverses this effect. B) Phalloidin staining: PANX2-OE induces F-actin contraction in H1299 cells (reversed by DTT) but not in BEAS-2B cells. C) Phalloidin and DiO co-staining: PANX2-OE induces disruption of cytoskeleton-membrane interactions in H1299 cells (reversed by DTT) but not in BEAS-2B cells. D) Correlation analysis of PANX2 and SLC7A11. E) qRT-PCR to evaluate SLC7A11 mRNA levels in PANX2-OE/KO cells vs. controls. F) Cystine uptake assay in PANX2-OE/KO cells vs. controls. G) Cystine level detection in PANX2-OE/KO cells vs. controls. H) NADP^+^/NADPH ratio analysis of NADPH consumption in PANX2-OE/KO cells vs. controls. I) WB to assess SLC7A11-KD or cystine starvation on PANX2-OE-upregulated SLC7A11 protein levels in H1299 cells. J) qRT-PCR to assess SLC7A11-KD on PANX2-OE-upregulated SLC7A11 mRNA levels in LUAD cells. K) Cystine level detection: SLC7A11-KD or cystine starvation -Cys reverses PANX2-OE-upregulated cystine levels in H1299 cells. L) NADP+/NADPH ratio analysis: SLC7A11-KD or -Cys reverses PANX2-OE-upregulated NADPH consumption in H1299 cells. M) Non-reducing SDS-PAGE: SLC7A11-KD or -Cys reversed PANX2-OE-induced disulfide crosslinking of cytoskeletal proteins in H1299 cells (Long exposure). N) Phalloidin staining: SLC7A11-KD or -Cys reversed PANX2-OE-induced F-actin contraction in H1299 cells.

Figure S4.

**Figure S4.** A) Correlation analysis of PANX2 and NRF2. B) qRT-PCR to evaluate NRF2 mRNA levels in PANX2-OE/KO cells vs. controls. C) WB time-course analysis of NRF2 protein stability upon PANX2-OE/KO in CHX-treated H1299 cells (40 μg/ml). D) qRT-PCR to evaluate NQO1 and HMOX1 mRNA levels in PANX2-OE/KO cells vs. controls. E) Ca^2+^ flux measurement to assess PANX2-OE/KO on intracellular Ca^2+^ flux and its reversal by BAPTA-AM (10 μM, 30 min) in LUAD cells. F) WB: BAPTA-AM (10 μM, 30 min) reverses PANX2-OE-upregulated NRF2 protein levels in LUAD cells. G) WB to assess the effect of NRF2-KD and subsequent SLC7A11 re-expression on PANX2-OE-upregulated NRF2 and SLC7A11 protein levels in H1299 cells. H) qRT-PCR to assess the effect of NRF2-KD and subsequent SLC7A11 re-expression on PANX2-OE-upregulated SLC7A11 mRNA levels in LUAD cells. I) Cystine level detection to assess the effect of NRF2-KD and subsequent SLC7A11 re-expression on PANX2-OE-upregulated cystine level in H1299 cells. J) NADP+/NADPH ratio analysis to assess the effect of NRF2-KD and subsequent SLC7A11 re-expression on PANX2-OE-upregulated NADPH consumption in H1299 cells. K) Non-reducing SDS-PAGE assay to assess the effect of NRF2-KD and subsequent SLC7A11 re-expression on PANX2-OE-induced disulfide crosslinking of cytoskeletal proteins in H1299 cells. L) Phalloidin staining assay to assess the effect of NRF2-KD and subsequent SLC7A11 re-expression on PANX2-OE-induced F-actin contraction in H1299 cells.

Figure S5.

**Figure S5.** A) Correlation analysis between PANX2 and SLC2A1, SLC2A3, G6PD. B) qRT-PCR to evaluate SLC2A1 mRNA levels in PANX2-OE/KO cells vs. controls. C) qRT-PCR to evaluate SLC2A3 mRNA levels in PANX2-OE/KO LUAD cells vs. controls. D) WB to evaluate SLC2A1 and SLC2A3 protein levels in PANX2-OE/KO LUAD cells vs. controls. E) Glucose uptake assay in PANX2-OE/KO LUAD cells vs. controls. F) qRT-PCR to evaluate G6PD mRNA levels in PANX2-OE/KO cells vs. controls. G) G6PD activity assay in PANX2-OE/KO cells vs. controls. H) WB to assess the effect of G6PD-OE on PANX2-OE-downregulated G6PD protein levels in H1299 cells. I) qRT-PCR to assess the effect of G6PD-OE on PANX2-OE-downregulated G6PD mRNA levels in LUAD cells. J) Cystine level detection: G6PD-OE reverses PANX2-OE-upregulated cystine levels in H1299 cells. K) NADP+/NADPH ratio analysis: G6PD-OE reverses PANX2-OE-upregulated NADPH consumption in H1299 cells. L) Non-reducing SDS-PAGE: G6PD-OE reversed PANX2-OE-induced disulfide crosslinking of cytoskeletal proteins in H1299 cells. M) Phalloidin staining: G6PD-OE reversed PANX2-OE-induced F-actin contraction in H1299 cells. N) WB to assess the effect of NRF2-KD on PANX2-OE-downregulated G6PD protein levels in LUAD cells. O) CCK-8 assay to evaluate the rescue effect of different reagent combinations on PANX2-OE-induced viability loss in LUAD cells. P) CCK-8 assay to evaluate the effect of different protein expression interventions on cell viability, mimicking PANX2-OE-induced effects, in LUAD cells.

Figure S6.

**Figure S6.** A) ssGSEA analysis of immune cell infiltration in GSE40275 lung cancer: 28 cell types with differential infiltration scores between high/low-PANX2 expression groups. B) Schematic of the construction of LUAD-immune cell co-culture model. C) Co-culture microscopy: immune cell migration and aggregation from PANX2-OE/KO co-cultures vs. controls. D) ELISA of cytokines/chemokines in co-culture supernatants from PANX2-OE/KO co-cultures vs. controls. E) Flow cytometry immunophenotyping: percentage of NK cells (CD56^+^) in CD3^-^ lymphocytes in PANX2-OE/KO co-cultures vs. controls. F) Flow cytometry: proportion of cytotoxic NK cells subset (CD56dimCD16^+^) to secretory NK cells subset (CD56briCD16^-^) in PANX2-OE/KO co-cultures vs. controls. G) Flow cytometry: proportion of DC cells (CD11c^+^) in total monocytes in PANX2-OE/KO co-cultures vs. controls. H) Flow cytometry: percentage of B cells (CD19^+^) in CD3^-^ lymphocytes in PANX2-OE/KO co-cultures vs. controls. I) Flow cytometry: proportion of M1 macrophages (CD86^+^CD206^-^) in CD14^+^ monocytes-derived macrophages and M1/M2 ratio (CD86^+^CD206^-^/CD206^+^CD86^-^) in PANX2-OE/KO co-cultures vs. controls. J) Chemiluminescence assay of extracellular ATP release in PANX2-OE/KO cells vs. controls. K) CCK-8 assay: A438079 (10 μM, 72 h) does not affect A549 cell viability.

Figure S7.

**Figure S7.** A) Schematic of humanized mouse model with subcutaneous LUAD xenografts. B) Flow cytometry: hCD45+ cell percentages in mouse peripheral blood 1 week after hPBMC transfer (40–60%). C) WB of PANX2/NRF2/SLC7A11/G6PD protein levels in tumor samples from PANX2-OE/KO groups vs. controls. D) qRT-PCR of PANX2/NRF2/SLC7A11/G6PD mRNA levels in tumor samples from PANX2-OE/KO groups vs. controls. E) WB to assess the effect of the indicated expression interventions on NRF2, SLC7A11, and G6PD protein levels in tumor tissues from PANX2-OE xenografts. F) qRT-PCR to assess the effect of the indicated expression interventions on SLC7A11 and G6PD mRNA levels in tumor tissues from PANX2-OE xenografts. G) Cystine level assay in PANX2-OE xenografts with the indicated protein expression interventions vs. PANX2-OE alone and non-PANX2-OE controls. H) NADP+/NADPH ratio analysis to assess NADPH consumption in PANX2-OE xenografts with the indicated protein expression interventions vs. PANX2-OE alone and non-PANX2-OE controls. I) H&E staining to assess cancer cell death in tumor tissues from PANX2-KO xenografts vs. controls. J) H&E staining to assess immune infiltration and cancer cell death in tumor tissues from PANX2-OE xenografts with A740003 treatment vs. PANX2-OE alone and non-PANX2-OE controls, and from PANX2-KO xenografts vs. controls. K) mIF to visualize immune infiltration in tumor tissues from PANX2-KO xenografts vs. controls.

Figure S8.

**Figure S8.** A,B) Drug sensitivity analysis: comparison of predicted drug sensitivity between PANX2-high/low lung cancers (top 20 drugs with significant differences shown).

Figure S9.

**Figure S9.** A) Gating strategy for flow cytometric immunophenotyping. B) Color-coding scheme for flow cytometric immunophenotyping. C,D) mIF targets and color schemes.

Table S1.

**Table S1.** Clinical characteristics of the GSE40275 dataset used for PANX2 expression analysis across normal and tumor tissues, and by clinical stage.

Table S2.

**Table S2.** Clinical follow-up and overall survival data of the HPA LUAD cohort used for survival analysis stratified by PANX2 expression.

Table S3.

**Table S3.** Clinical follow-up and prognostic data of the TCGA LUAD cohort used for DFI and PFI analyses stratified by PANX2 expression.

Table S4.

**Table S4.** List of primary antibodies used for WB in this study, including source, catalog number, and dilution.

Table S5.

**Table S5.** Primer sequences used for qRT-PCR in this study.

Table S6.

**Table S6.** GSEA results comparing high vs. low PANX2 expression groups in the GSE40275 dataset, including enriched GO terms and KEGG pathways.

Table S7.

**Table S7.** Pearson correlation analysis between PANX2 and disulfidptosis-related genes in the GSE40275 dataset.

Table S8.

**Table S8.** ssGSEA scores for 28 immune cell types in high vs. low PANX2 expression groups from the GSE40275 dataset.

Table S9.

**Table S9.** Predicted drug sensitivity for 198 compounds in high vs. low PANX2 expression groups using the oncoPredict algorithm (GDSC2 model) based on the GSE40275 dataset.
