# Supplementary figures and images for: PANX2 Suppresses Lung Adenocarcinoma Progression by Inducing Disulfidptosis and Enhancing Antitumor Immunity
Source: Adv Sci (Weinh). 2026 May 29:e75662. Online ahead of print. doi: 10.1002/advs.75662 (PMC13335910; doi:10.1002/advs.75662)

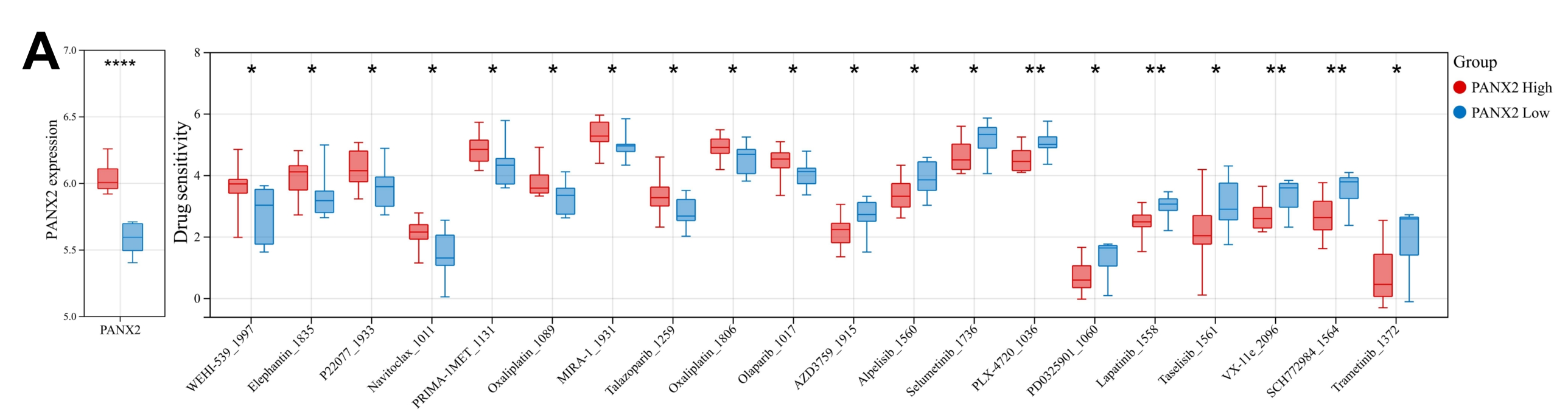

Supplement: Supplementary file 2 — Supporting File 2: advs75662‐sup‐0002‐FiguresS1‐S9.zip. [file ADVS-9999-e75662-s002.zip › Figure S8, Supporting Information.png]
